# Quantitative Trait Nucleotides Impacting the Technological Performances of Industrial *Saccharomyces cerevisiae* Strains

**DOI:** 10.3389/fgene.2019.00683

**Published:** 2019-07-23

**Authors:** Emilien Peltier, Anne Friedrich, Joseph Schacherer, Philippe Marullo

**Affiliations:** ^1^Department Sciences du vivant et de la sante, Université de Bordeaux, UR Œnologie EA 4577, Bordeaux, France; ^2^Biolaffort, Bordeaux, France; ^3^Department Micro-organismes, Génomes, Environnement, Université de Strasbourg, CNRS, GMGM UMR 7156, Strasbourg, France

**Keywords:** biotechnology, fermentation, QTL, QTN, QTG, yeast, variant, aroma

## Abstract

The budding yeast *Saccharomyces cerevisiae* is certainly the prime industrial microorganism and is related to many biotechnological applications including food fermentations, biofuel production, green chemistry, and drug production. A noteworthy characteristic of this species is the existence of subgroups well adapted to specific processes with some individuals showing optimal technological traits. In the last 20 years, many studies have established a link between quantitative traits and single-nucleotide polymorphisms found in hundreds of genes. These natural variations constitute a pool of QTNs (quantitative trait nucleotides) that modulate yeast traits of economic interest for industry. By selecting a subset of genes functionally validated, a total of 284 QTNs were inventoried. Their distribution across pan and core genome and their frequency within the 1,011 *Saccharomyces cerevisiae* genomes were analyzed. We found that 150 of the 284 QTNs have a frequency lower than 5%, meaning that these variants would be undetectable by genome-wide association studies (GWAS). This analysis also suggests that most of the functional variants are private to a subpopulation, possibly due to their adaptive role to specific industrial environment. In this review, we provide a literature survey of their phenotypic impact and discuss the opportunities and the limits of their use for industrial strain selection.

## Introduction

Between individuals of the same species, a broad palette of genetic variants is found, including large chromosomal rearrangements (deletions, duplications, inversions, translocations, and introgressions) and punctual mutations (Griffiths et al., 2000). This latter type includes small insertions/deletions (InDels) as well as single-nucleotide polymorphisms (SNPs) that are by far the most frequent polymorphic event found at the intraspecific level in fungi (Doniger et al., 2008), human (Sachidanandam et al., 2001), and plants (Ching et al., 2002). Depending on the organism and the genomic position, the SNP/InDel frequency ranges from 1×10^−2^ to 1×10^−3^ per base and constitutes a vast pool of genetic variants (Stucki et al., 2012; Almeida et al., 2014; Scozzari et al., 2014; Peter et al., 2018).

With the relative ease of obtaining genome-wide SNP-data, their impact on complex trait can be tracked by either genome-wide association studies (GWAS) or quantitative trait loci mapping (QTL mapping) in medicine (Beck et al., 2014) or agronomy (Brachi et al., 2011; Sharma et al., 2015). When they are statistically linked to a phenotype, these SNPs become QTNs (quantitative trait nucleotides) and could be listed in large databases for research communities (Grant et al., 2010; Youens-Clark et al., 2011). In contrast to multicellular eukaryotes, large SNP-database regrouping several studies for fungi and yeasts are not really developed. For *Saccharomyces cerevisiae* species, the first attempts to set up SNP databases have been made 10 years ago (Doniger et al., 2008; Schacherer et al., 2009) but without establishing a link between genotypic data and phenotypes.

With the emergence of high-throughput sequencing in the last 10 years, the number of available complete genomes rose impressively providing a quite complete landscape of genetic polymorphism for this species (Peter et al., 2018). A particular focus was done on strains belonging to food fermentation including wine (Borneman et al., 2016), beer (Gallone et al., 2016), distillery (Barbosa et al., 2018), and cheese/flor/distillery (Legras et al., 2018). As previously demonstrated, the *S. cerevisiae* population appears to be clearly structured according to the geography, the environmental niche, and the relation to human environment (Peter and Schacherer, 2016; Marsit et al., 2017; Peter et al., 2018). Among the food-related strains, the beer and bakery strains are polyphyletic and characterized by a high ploidy level (Gallone et al., 2016), whereas wine or sake strains are mostly diploid and derived from the genetic drift of a limited number of founders (Ohnuki et al., 2017). Since each industrial application is characterized by distinct populations, the strains of each group have been faced with specific selective pressures. These conditions have likely promoted the emergence of adaptive alleles conferring a phenotypic advantage to each particular industrial process. The identification of those adaptive mutations in the wide pool of natural variations that discriminate the different subpopulations remains a challenging task. It has been recently shown by GWAS that CNVs (copy number variants) and gross chromosomal reorganization exert a sound effect on phenotypic variation (Peter et al., 2018). However, the yeast strains of each subpopulation also exhibit a wide set of SNPs shaping their technological properties. The growing number of QTNs found in the last decade suggests that numerous functional variants will be found in the future.

In this review, we established an extensive catalog of *S. cerevisiae* QTNs experimentally validated that impact traits of biotechnological interest. First, we analyzed their allelic frequencies and their dispersion within a large population. Second, we reported their physiological effect. Third, we discussed how these QTNs can be used for significantly improving the technological properties of industrial yeast strains.

## Genes and Polymorphisms Impacting Quantitative Traits of Industrial Interest

**Figure 1:**
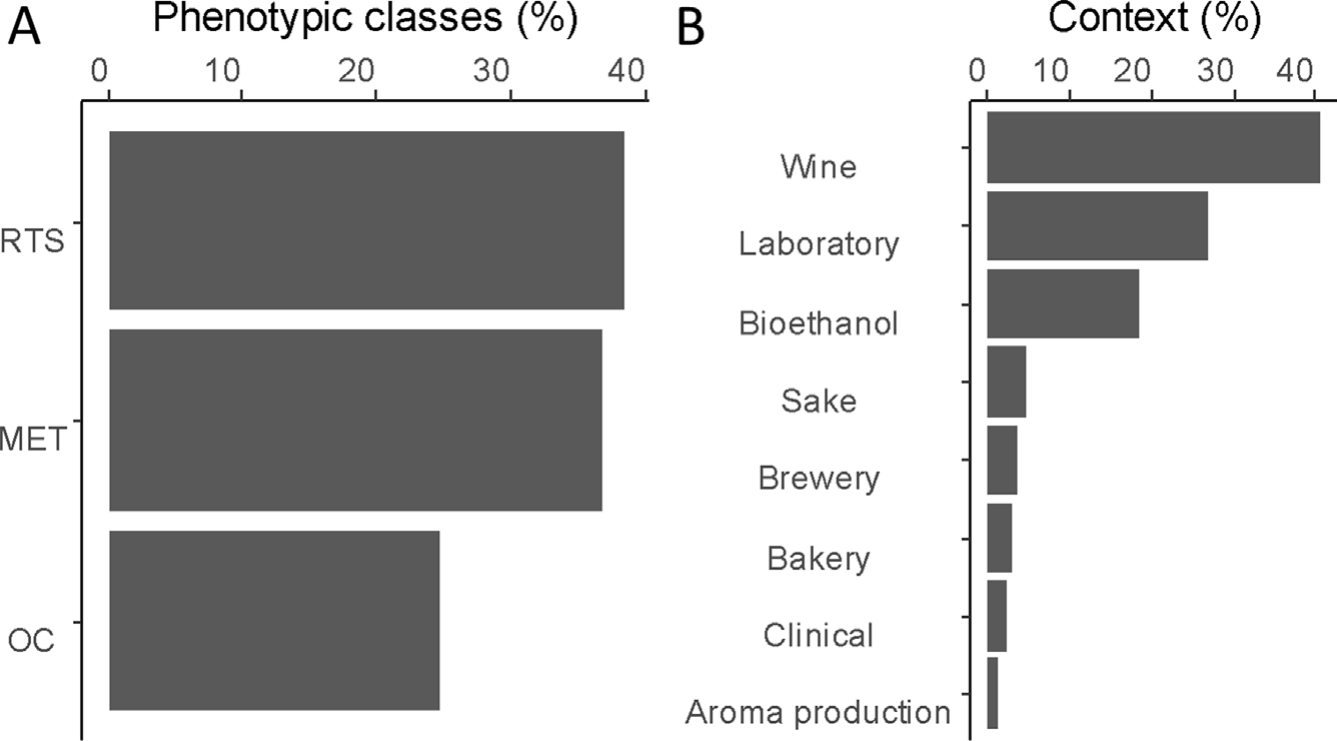
Overview of identified quantitative trait genes (QTGs). **(A)** Proportion of genetic variants according to three main categories. Resistance to stress (RTS), central metabolism (MET), and organoleptic compound (OC). (**B)** Proportion of QTGs according to the context of the study in which they were identified.

To establish an exhaustive catalog of functional variants, we focus our literature survey on QTNs impacting yeast traits relevant for biotechnological applications. Traits were sorted in three main phenotypic classes: traits linked to metabolism (e.g., nitrogen, carbon, vitamin, and fermentation activity), traits linked to stress resistance (e.g., acidic and basic, temperature, osmotic, and ethanol), and traits impacting the organoleptic properties of the products (**Figure 1A**). Most of QTNs were identified by linkage analysis, demonstrating the efficiency of this strategy in yeast (Liti and Louis, 2012; Fay, 2013). Other functional variants were identified by mutagenesis, comparative genomics, and adaptive laboratory evolution (ALE) approaches (Gresham and Hong, 2014). More recently, genome-wide associations were performed on an extensive set of fully sequenced natural isolates, providing a large list of SNPs statistically associated with the measured phenotypic diversity (Peter et al., 2018; Sardi et al., 2018).

**Figure 2:**
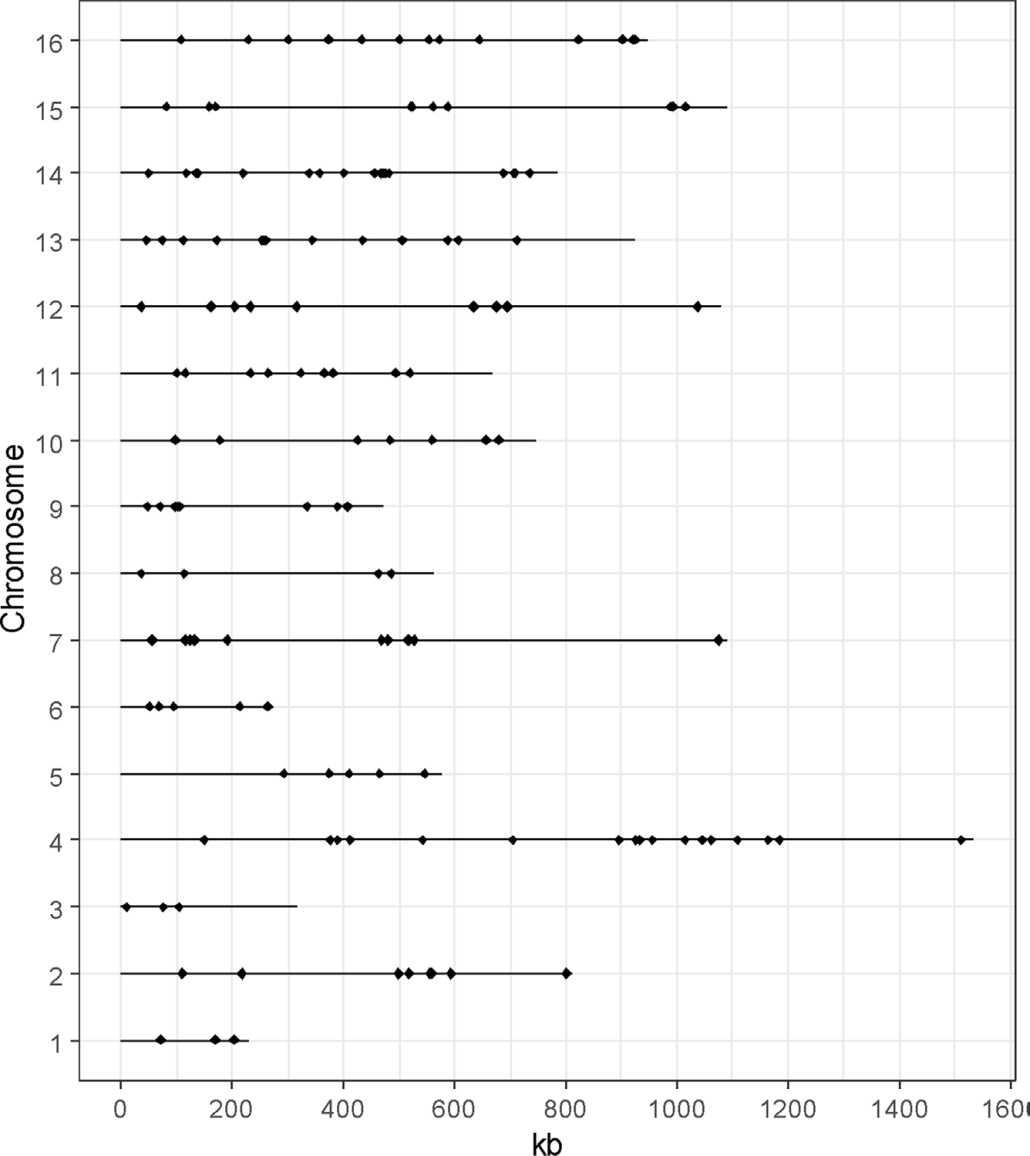
Distribution of the QTGs along the genome. Each dot represents the position of one QTG. No QTG hotspot was found (hypergeometric distribution, 1,000 permutations test, FDR = 5%).

To provide a functional analysis, we included only genes that have been experimentally validated by reciprocal hemizygosity assay (RHA) (Steinmetz et al., 2002) or allele swapping (Storici and Resnick, 2006). In this context, a total of 147 QTGs (quantitative trait genes) were reported and described across a set of 85 articles (**Table S1**). In total, 71% of these genes were identified in an industrial context since they concern media and/or strains related to aroma production (1%), bioethanol (18%), or traditional fermented goods including wine (41%), sake (5%), bakery (3%), or beer (3%). The remaining 30% were identified by using laboratory or clinical strains cultivated in non-industrial conditions (**Figure 1B**). However, these genes were included since they possibly affect industrial properties [e.g., growth fitness, stress resistance, and flocculation). We tested the overall distribution of such genes across yeast genome, and no hotspot was found (hypergeometric distribution, with sliding windows of 100 kb and a 10-kb step, 1,000 permutation tests, False Discovery Rate (FDR) = 5%](**Figure 2**).

**Figure 3:**
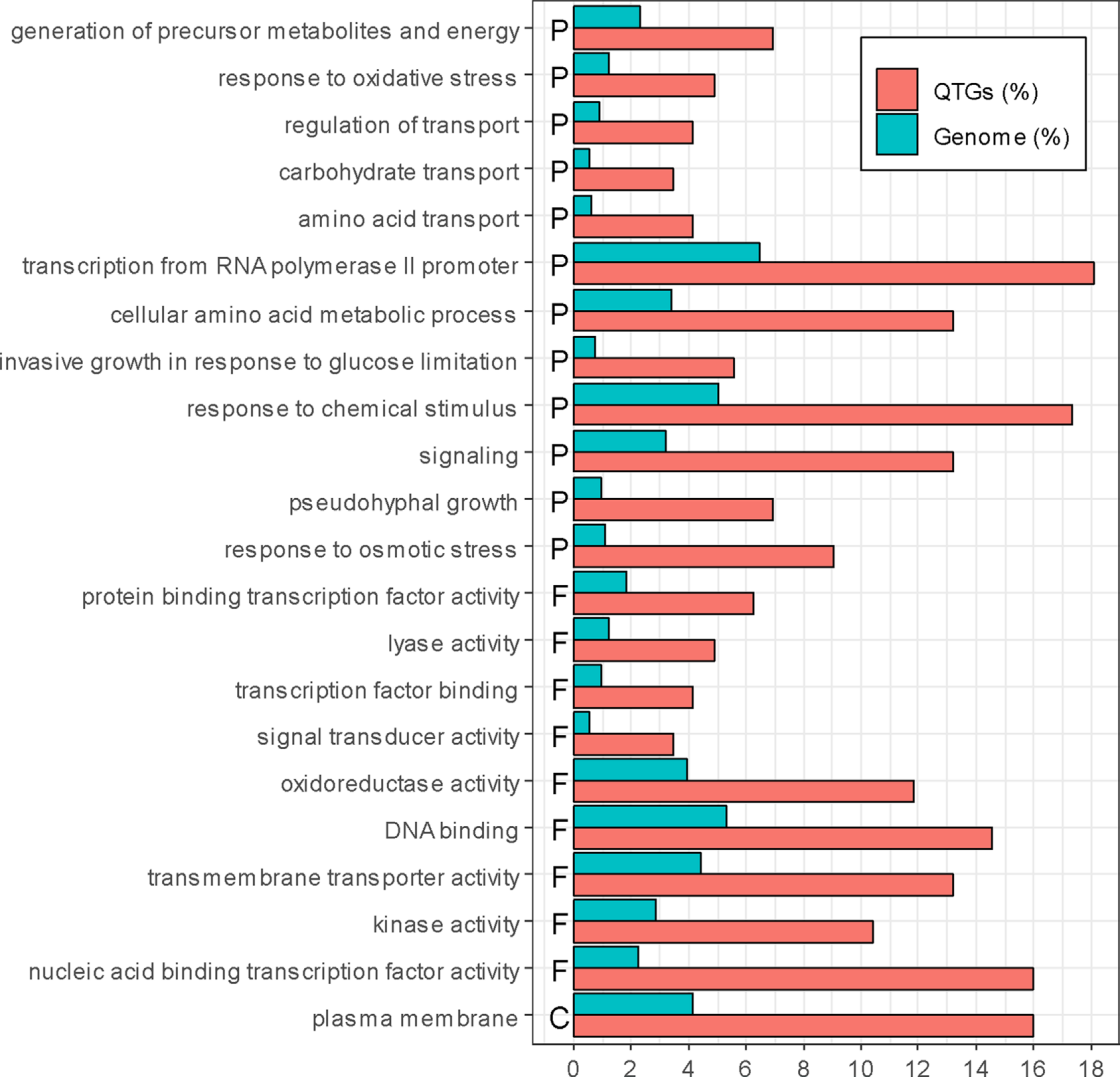
Enrichment of QTGs. Only significantly over-represented or under-represented categories are represented (chi-squared test, *p*-value < 0.05, with Bonferroni adjustment for multiple tests). The percentage of genes in the genome and in the QTGs is indicated in blue and red, respectively. F, P, and C represent molecular function, biological process, and cellular component, respectively.

Among these 147 QTGs, a significant enrichment was obtained for Gene Ontology terms (goTermFinder, https://www.yeastgenome.org) (**Figure 3**). This is the case for function and process terms related to transcription (nucleic acid-binding transcription factor activity, DNA binding, protein-binding transcription factor activity, and transcription factor binding) (*p*-value < 0.05) and transport (plasma membrane, transmembrane transporter activity, regulation of transport, amino acids transport, and carbohydrate transport) (*p*-value < 0.05). The strong enrichment of such categories confirms that these genes are important levers for generating phenotypic variability.

**Figure 4:**
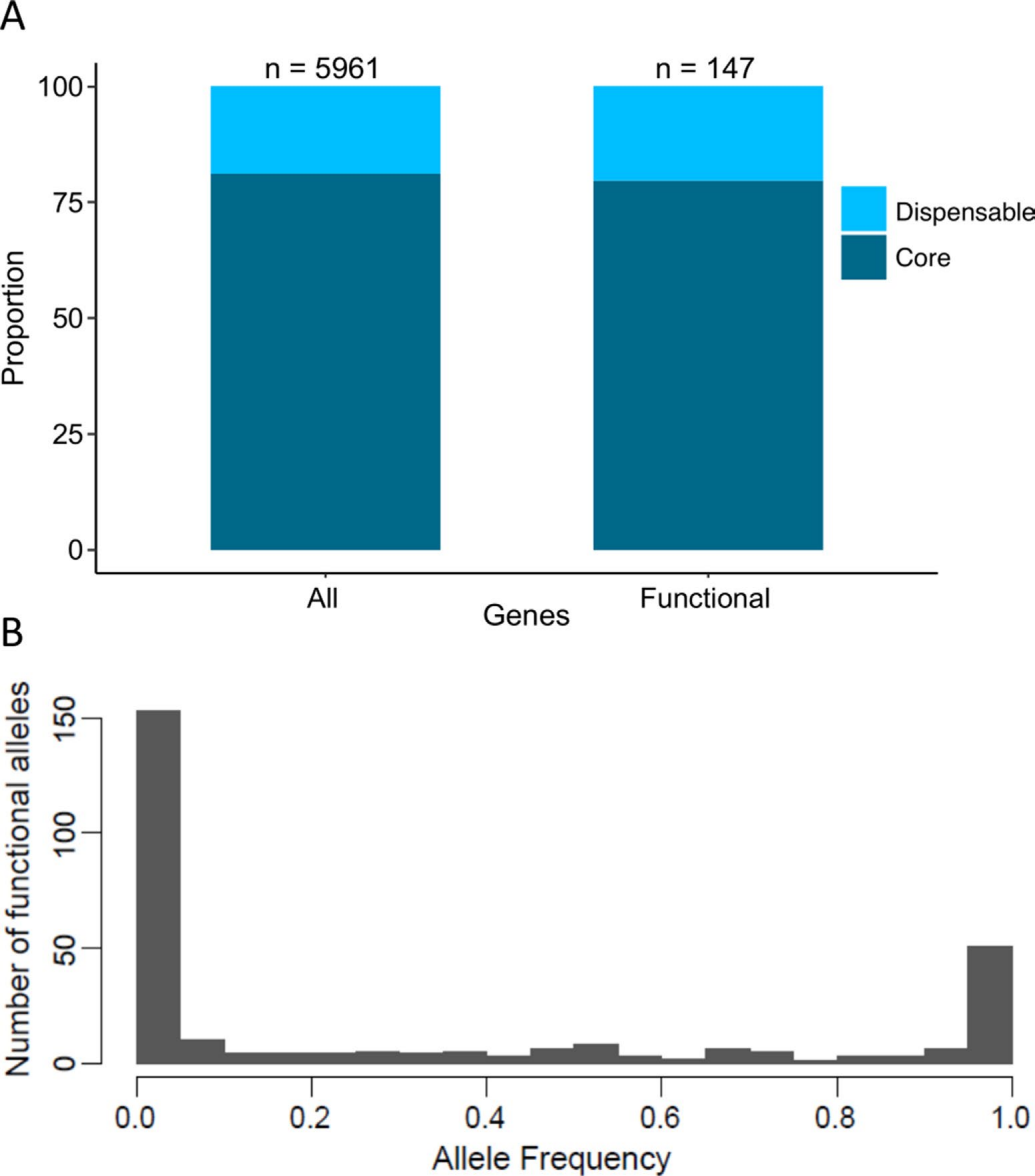
Proportion of dispensable/core gene and frequency of functional alleles. **(A)** Dispersion of QTGs allele frequency among 1,011 isolates. For each QTG, the frequency of the favorable alleles is used. **(B)** Proportion of dispensable and core genes among the pangenome (all) and among QTGs (functional).

In *S. cerevisiae*, the pangenome was recently defined using a population of 1,011 natural isolates. Overall, 4,940 core genes and 2,856 accessory genes were determined within the population (Peter et al., 2018). Interestingly, a strong enrichment has been shown for genes whose function is related to adaptation to the environment in the subset of accessory genes (Peter et al., 2018). Across the 147 identified QTGs, 117 and 30 are part of the core and accessory genomes, respectively (**Figure 4A**). This proportion clearly shows that a large fraction of the QTGs are part of the conserved core genome. Moreover, there is no bias toward the subset of accessory genes, although they are more prone to be involved to adaptation processes.

In order to focus our review at the SNP level, we listed all the possible genetic polymorphisms found in these QTGs. Since most of the articles validated genes but not at the SNP level, we took into account for each QTG all the genetic polymorphisms described by the authors. These 284 QTNs can be sorted in several categories according to the type of genetic polymorphisms that discriminate their allelic variations. Most of the identified allelic variations correspond to one or a combination of missense mutations, also called non-synonymous substitutions (nsSNPs) (81%). Other minor cases correspond to SNPs or InDels in 5′UTR or 3′UTR regions (6%), InDels in the coding sequence (6%), synonymous SNPs (sSNPs) (4%), translocation (2%), and short tandem repeats.

The functional impact of a subset of 251 QTNs, corresponding to missense mutations, was estimated by using the predictive program SIFT (sorting tolerant from intolerant) (Ng and Henikoff, 2003). It turns out that 168 mutations are predicted to be tolerated, whereas only 83 are predicted to be deleterious, suggesting that most of the QTNs do not lead to a loss of function (**Table S2**). By contrast, 22 QTNs (approximately 8%) correspond to nonsense mutations, meaning that genetic variant results in a premature stop codon generating in most of the cases to a loss of function. In addition, one and two QTNs lead to the loss of start and stop codons, respectively.

One main feature of the *S. cerevisiae* species is the bias toward low-frequency variant. Among the 1,011 recently sequenced genomes, much of the detected genetic polymorphisms are very low-frequency variants with 93% of them having a minor allele frequency lower than 0.1 (Peter et al., 2018). This observation raises the question regarding the impact of these variants on the phenotypic diversity. Among the 284 QTNs, more than 150 have a frequency lower than 5%, meaning that these variants would be undetectable by GWAS (**Figure 4B**).

Population genomic studies in *S. cerevisiae* also allowed to define precisely the different subpopulations, which are related to either the ecological or geographical origins. Based on the 1,011 isolates, a total of 26 subpopulations were defined (Peter et al., 2018). Interestingly, the identified QTNs are not evenly distributed across these subpopulations, and biases toward some specific of them are observed (**Table S3**). For example, 26 QTNs are private to the wine subpopulation and are only found in 9 to 50 wine isolates. Two SNPs located the *MSN2* and *MSN4* genes are exclusively found in the sake subpopulation. And finally, a mutation in the *MAL33* gene is private to the African beer population. This observation clearly suggests that most of the functional variants are private to a subpopulation. This could result to the adaptation to a specific industrial environment. However, since wine and sake subpopulations are derived from a limited number of founders, their presence could be also explained by simple genetic drift.

Overall, this set of 284 QTNs is very insightful but does not reflect the genetic architecture in its entirety. The genotypic landscape is not limited to SNPs located in protein coding regions. Indeed, SNPs as well as InDels located in promoter and 3′ untranslated regions were identified for some complex traits. These genetic variants do not affect the protein sequence but can impact the transcription level, mRNA processing, translation, export, and decay. Mutations altering a functional motif in the promoter region have been identified many times (Shahsavarani et al., 2012; Zimmer et al., 2014; Cubillos, 2016; Salinas et al., 2016; Tapia et al., 2018). They constitute allele-specific expression (ASE) changes that are certainly a source of phenotypic variation (see Cubillos, 2016, for a review). In addition, structural variants such as CNVs and translocations were identified as involved in the variation of some specific industrial traits. Recently, genome-wide association analyses, performed on a large collection of *S. cerevisiae* isolates, highlighted the importance of the CNVs, which explain a more considerable proportion of the phenotypic variance and have greater effects on phenotype compared with the SNPs (Peter et al., 2018). However, the detection of the structural variants at a population scale is yet technically limited, and consequently, their global impact on the complex traits is still to be explored.

## Industrial Traits Impacted by Natural Genetic Variants

In this section, we shortly described the phenotypic impact of most of QTGs/QTNs reviewed. The phenotypes surveyed were arranged in three main subsections concerning central metabolism, resistance to toxins and stresses, and organoleptic contribution.

### Central Metabolism

In total, 71 natural alleles have been identified, and they impact 70 traits related to the central metabolism (**Table 1** and **Table S1**).

**Table 1:** Nature of SNP grouped by phenotypes.

Phenotype categories	Genes involved (experimentally validated)	Number and nature of the molecular cause involved							No data	References
		Total	nsSNP	InDels	5′ or 3′UTR positions	Translocation	STR	sSNP		
Central metabolism										
Nitrogen requirement, growth rate on specific nitrogen sources, ammonium and amino acid uptake, vitamin biosynthesis	*GCN1*, *DAL1*, *DAL4*, *LST4*, *PUT4*, *GAT1*, *MEP2*, *ABZ1*, *ALP1*, *ARO1*, *ASI2*, *CPS1*, *LYP1*, *PDC1*, *RIM15*, *ASN1*, *AGP1*, *BUL2*, *GLT1*, *ADE5*, *ARO8*, *VBA3 ASI1*, *ARG81*, *BIO3*, *MDS3*, *ASP1*	29	18	2	2	0	0	0	7	(Omura et al., 2005; Marullo et al., 2007a; Ambroset et al., 2011; Kwan et al., 2011; Gutiérrez et al., 2013; Hong and Gresham, 2014; Brice et al., 2014; Ibstedt et al., 2014; Jara et al., 2014; Salinas et al., 2016; Cubillos et al., 2017)
Sugar catabolism, fructose, glucose, maltose, and maltotriose uptake, glucose–galactose switch, diauxic switch	*HXT3*, *MTH1*, *GAL80*, *MAL33*, *MAL11*, *HAP4*, *MBR1*, *ADR1*, *BMH1*, *YJR030c*	10	10	0	0	0	0	0	0	(Higgins et al., 1999; Segrè et al., 2006; Guillaume et al., 2007; Vidgren et al., 2009; Kvitek and Sherlock, 2011; Salinas et al., 2012)
Glycerol metabolism	*SSK1*, *GPD1*, *HOT1*, *SMP1*, *GUT1*, *GAT1*, *YFL040W*, *TAO3*, *ADH3*	10	7	1	0	0	0	0	2	(Salinas et al., 2012; Hubmann et al., 2013a; Hubmann et al., 2013b; Swinnen et al., 2013; Wilkening et al., 2014; Tapia et al., 2018)
Acetic acid production	*YAP1*, *FAS2*, *ASP1*, *ALD6*	4	3	0	1	0	0	0	0	(Inokoshi et al., 1994; Marullo et al., 2007a; Salinas et al., 2012; Cordente et al., 2013)
Fermentation rate and completion, *fil* phenotype	*CYR1*, *GPR1*, *HAP4*, *MBR1*, *YJR030c*, *ABZ1*, *GDB1*, *MSB2*, *PDR1*, *PMA1*, *VMA13*, *PDR3*, *SSU1*, *MSN2*, *MSN4*, *RIM15*, *ADR1*	18	15	1	1	1	0	0	0	(Mizoguchi et al., 2002; Ambroset et al., 2011; Watanabe et al., 2011; Salinas et al., 2012; Watanabe et al., 2013; Cubillos, 2016; Martí-Raga et al., 2017; Peltier et al., 2018)
Resistance to toxins and stresses										
Ethanol accumulation capacity, ethanol tolerance, growth on ethanol	*ADE1*, *KIN3*, *SSD1*, *UTH1*, *VPS70*, *IAI11*, *APJ1*, *MKT1*, *SWS2*, *MEX67*, *PRT1*, *VPS70*, *SPT5*, *TAO3*	14	12	0	2	0	0	0	O	(Yang et al., 2011; Avrahami-Moyal et al., 2012; Salinas et al., 2012; Swinnen et al., 2012b; Hubmann et al., 2013a; Hubmann et al., 2013b; Pais et al., 2013; Duitama et al., 2014; Wilkening et al., 2014; Voordeckers et al., 2015)
High-temperature growth, temperature tolerance, low temperature adaptation, freezing tolerance	*CDC19*, *IRA1*, *IRA2*, *RSP5*, *END3*, *MKT1*, *RHO2*, *NCS2*, *TAO3*, *PRP42*, *SMD2*, *GAA1*, *FPK1*, *OPT2*, *PET494*, *QCR2*, *OYE2*, *VHS1*	18	14	0	2	0	0	1	1	(Sinha et al., 2006; Sinha et al., 2008; Parts et al., 2011; Benjaphokee et al., 2012; Shahsavarani et al., 2012; Li et al., 2013; Wilkening et al., 2014; García-Ríos et al., 2017; Marullo et al., 2019)
Stress tolerance, oxidative, osmotic	*PRO1*, *MPR1*, *AQY1*, *AQY2*, *RCK2*, *MOT2*, *MPR1*, *RDS2*, *PUT1*, *SPT5*	12	8	4	0	0	0	0	0	(Sekine et al., 2007; Takagi, 2008; Will et al., 2010; Diezmann and Dietrich, 2011; Dhar et al., 2011; Sasano et al., 2012; Greetham et al., 2014)
Toxins resistance: acidic, basic, phenolic compounds, SO_2_	*WAR1*, *ADH3*, *ASG1*, *GIS4*, *SKS1*, *COX20*, *CUP2*, *DOT5*, *GLO1*, *VMA7*, *HAA1*, *CDC23*, *ECM21*, *GPH1*, *IES2*, *MAC1*, *NMD4*, *SGT2*, *SPS100*, *LEU3*, *MNE1*, *SAP190*, *ADH1*, *MKT1*, *RAD5*, *UBP7*, *SSU1*	31	12	0	4	2	0	3	11	(Pérez-Ortín et al., 2002; Boaz et al., 2008; Demogines et al., 2008; Romano et al., 2010; Yang et al., 2011; Brion et al., 2013; Zimmer et al., 2014 Pinel et al., 2015; González-Ramos et al., 2016; Meijnen et al., 2016; Swinnen et al., 2017; Sardi et al., 2018)
Organoleptic compounds production										
Acids, higher alcohols, and relative ester production	*IXR1*, *LEU9*, *RGS2*, *RGT1*, *FAS2*, *ABZ1*, *TOR1*, *NRG1*, *FLX1*, *MDH2*, *AGP1*, *ALP1*, *ILV6*, *MAE1*, *AGP2*, *FAS1*, *SIR2*, *EAT1*, *SNF8*	19	19	0	0	0	0	0	0	(Akada et al., 1999; Salinas et al., 2012; Steyer et al., 2012; Trindade de Carvalho et al., 2017; Eder et al., 2018)
Sulfur compounds	*MET10*, *MET5*, *MET2*, *IRC7*, *URE2*, *SKP2*, *MET6*, *CYS4*, *SSU1*	9	5	1	0	1	0	0	2	(Cordente et al., 2009; Linderholm et al., 2010; Huang et al., 2014; Noble et al., 2015)
Other aromas	*PAD1*, *PDR8*, *ERG20*	3	3	0	0	0	0	0	0	(Chambon et al., 1991; Steyer et al., 2013)
Adhesion, flocculation, clumpiness	*FLO1*, *SFL1*, *GPA1*, *AMN1*, *RGA1*, *CDC28*, *FLO8*, *END3*, *IRA2*, *MSS11*, *TRR1*, *FLO11*	17	13	0	0	0	3	1	0	(Yvert et al., 2003; Verstrepen et al., 2005; Fidalgo et al., 2006; Nogami et al., 2007; Yang et al., 2011; Li et al., 2013; Taylor and Ehrenreich, 2014; Wilkening et al., 2014)

#### Nitrogen and Vitamin Metabolism

Nitrogen sources as well as the vitamin composition can vary in a wide range according to the raw vegetal material, the fertilization method, and the harvest date. Their composition may drastically affect the yeast fermentation performances in beer (Gibson et al., 2007), wine (Bell and Henschke, 2005), or bio-ethanol productions (Hahn-Hägerdal et al., 2005). Depending on the genetic background, the strain’s ability to use various nitrogen sources differs between subpopulations (Ibstedt et al., 2014) and within strains of the same industrial process (Jiranek et al., 1995; Manginot et al., 1998; Gutiérrez et al., 2013; Brice et al., 2014).

The identification of genetic factors controlling nitrogen consumption has been achieved by many QTL mapping studies (Ambroset et al., 2011; Brice et al., 2014; Ibstedt et al., 2014; Jara et al., 2014), one large-scale hemizygosity analysis (Gutiérrez et al., 2013), and one ALE experiment (Gresham and Hong, 2014). These studies revealed relevant genetic variants that could be used for improving the performance of fermenting yeast. In such studies, the diverse media employed consist of either a mixture of different nitrogen sources, mimicking a natural medium (Ambroset et al., 2011; Brice et al., 2014; Jara et al., 2014), or several distinct media containing each a single nitrogen source (Gutiérrez et al., 2013; Gresham and Hong, 2014; Ibstedt et al., 2014). When a single nitrogen source was used, the identified QTLs were chiefly due to deleterious mutations in genes involved in the pathway of the amino acid concerned (Ibstedt et al., 2014). In contrast, in mixed nitrogen media, the effects of the QTLs identified are more pleiotropic and impact a group of amino acids sharing the same biochemical structure (Jara et al., 2014).

Deleterious alleles, impairing the use of a particular nitrogen source, were identified for proline (*PUT4*), allantoin (*DAL1* and *DAL4*) (Ibstedt et al., 2014), and methionine (*ARO8*, *VBA3*, and *ADE5*,*7*) (Gutiérrez et al., 2013). Similar deleterious mutations were also found for asparagine (*ASP1*) (Marullo et al., 2007a) or for folic acid metabolism (*ABZ1*), having an impact on wine fermentation kinetics (Ambroset et al., 2011). These recessive mutations are rare and generally of poor interest because industrial practices require prototrophic strains. However, these alleles can be used as auxotrophic markers for achieving breeding programs in a non-GMO context (Steensels et al., 2014) as the *ura3* and *lys2* markers (Timberlake et al., 2011; Dufour et al., 2013).

More interestingly, three pleiotropic genes (*GLT1*, *ASI1*, and *AGP1*) impacting consumption of several amino acids were identified by measuring the consumption profile of amino acids (Jara et al., 2014). By implementing a multi-parental design (SGRP-X4), these authors identified four additional genes (*ASI2*, *CPS1*, *LYP1*, and *ALP1*) involved in the consumption of aromatic and basic amino acids. In the same study, the comparative RNA-seq profiling of extreme progeny clones allowed the identification of two additional genes (*PDC1* and *ARO1*) that influence the amino acid consumption in the wine fermentation (Cubillos et al., 2017). Following a similar strategy, the progenies of two enological strains were phenotyped for their fermentation capacity in a synthetic grape must containing a low assimilable nitrogen level (Brice et al., 2014). Four genes directly or indirectly linked with the nitrogen metabolism were identified (*BIO3*, *GCN1*, *ARG81*, and *MDS3*).

**Figure 5:**
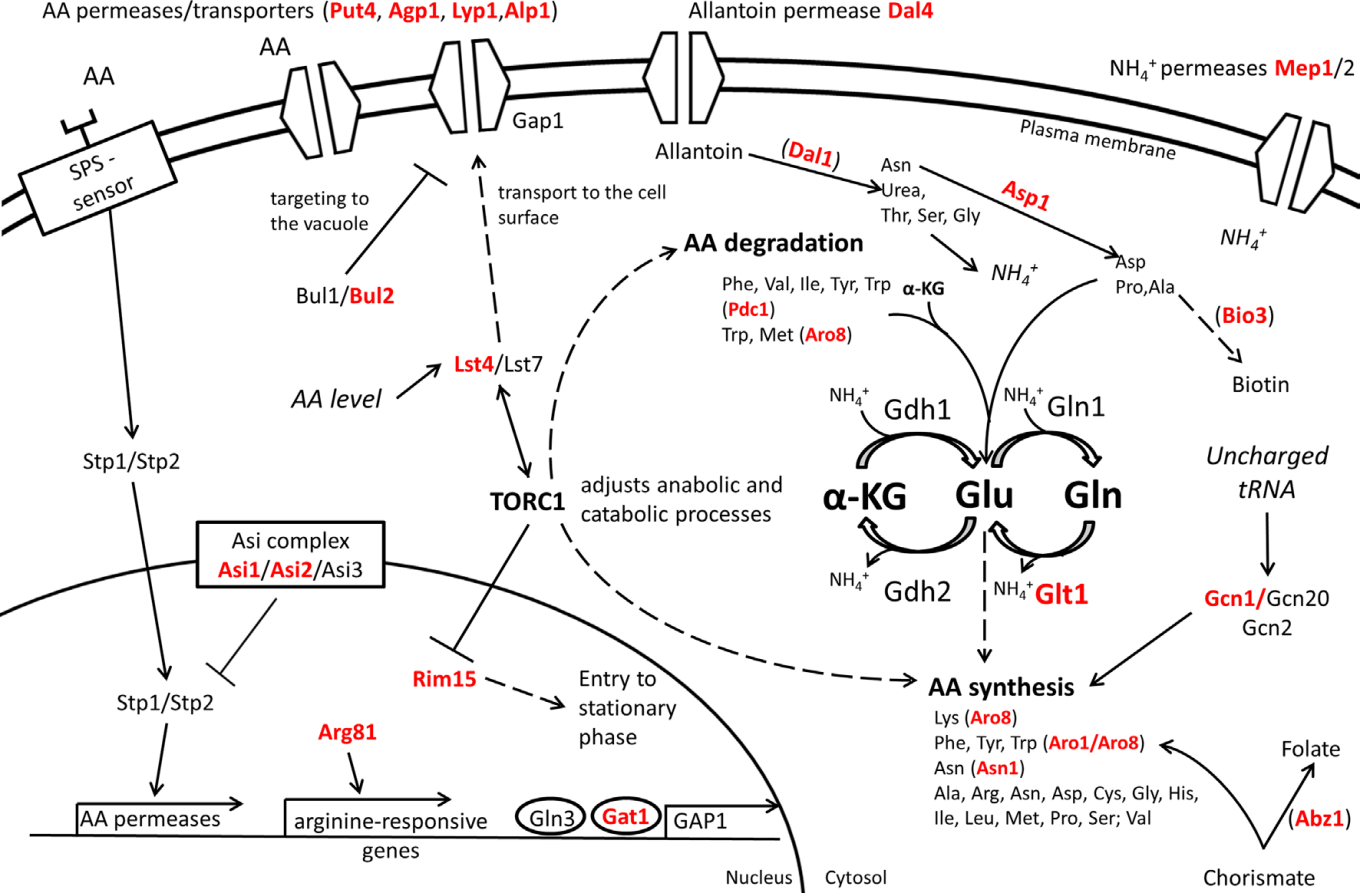
Overview of QTG involved in nitrogen metabolism. Proteins highlighted in red are coded by allelic variants that were experimentally validated for their contribution to variation in nitrogen source consumption in *S. cerevisiae*. Indirect relationships are shown in dashed lines. Proteins are between brackets when only the protein of interest of a pathway is shown.

The expression level and the stability of proteins involved in nitrogen catabolism may also contribute to nitrogen assimilation. For example, the ASE of *ASN1*, the asparagine synthetase, modulates the consumption of aspartic and glutamic acid in wine-related fermentations (Salinas et al., 2016). Amino acid assimilation, in particular for proline, can be also induced by increasing the half-life of the membrane transporter Put4p by changing some N-terminal arginine residues involved in its ubiquitination (Omura et al., 2005). Although the optimal consumption of nitrogen source is generally considered as a suitable technological trait, the rapid and complete consumption of amino acids may negatively affect fermentation capacities (Martí-Raga et al., 2015) and reduces the chronological life span (Kwan et al., 2011) in specific conditions. All together, these studies support that multiple molecular mechanisms impact the nitrogen assimilation including the nitrogen signaling pathways, metabolic enzymes, and protein degradation as summarized in **Figure 5**.

#### Sugar Catabolism

##### Control of Fermentative and Respiratory Switch

The expanded use of *S. cerevisiae* in biotechnology is likely due to its strong efficiency to dissimilate small sugars by both respiratory and fermentative routes (Pronk et al., 1996; Zampar et al., 2013). In the past, deleterious mutants unable to switch between fermentative and respiratory metabolism have been identified for *CAT8* (Zimmermann et al., 1977) and *ADR1* (Denis and Young, 1983). Besides these drastic mutations, the fermentation/respiration balance is controlled by many other genes, and considerable variations have been measured within strains and species (Quirós et al., 2014). Some alleles impacting this metabolic control have been identified in *HAP4* and *MBR1* (Salinas et al., 2012). Both genes are involved in mitochondrial function, and *HAP4* is a key transcription factor activating respiratory genes (Zampar et al., 2013). These particular alleles may partially activate the Krebs cycle, reducing the fermentation efficiency of yeast in winemaking conditions.

A similar alteration in fermentation/respiration balance was found in sake strains by a comparative transcriptomic approach (Watanabe et al., 2013). Two distinct loss-of-function alleles have been identified for *ADR1* in the sake strains K7 and K701. This transcription factor is activated when glucose becomes limiting (diauxic shift) and promotes ethanol catabolism by activating the transcription of *ADH2*. Therefore, strains lacking a functional *ADR1* might have an accelerated alcoholic fermentation in a sake-brewing context.

##### Sugar Uptake and Assimilation

Although *S. cerevisiae* is able to ferment many sugars, their uptake and catabolism obey priority rules. This regulation, called glucose catabolite repression, limits the speed and efficiency of the fermentation of many sugars (Gancedo, 1998). Industrial conditions offer selective constraints that have promoted the constitutive activation of non-preferred carbon sources uptake and catabolism.

An example is the fermentation of maltose and maltotriose, the two most abundant sugars in brewing wort (Stewart, 2006), which are also present in bakery doughs. The fermentation performance of beer strains is therefore defined by their capacity to transport those sugars. Depending on the species, i.e., *S. cerevisiae* (*ale* group) or *S. pastorianus* (*lager* group), different α-glucoside transporters have been described (Sc*AGT1*, *ScMALx1*, *SeMALx1*, *SeAGT1*, *MTT1*, and *ScMPHx*). Most of the ale beer strains have a full-length *AGT1* gene, which ensures complete wort fermentation. By contrast, the lager strains have a premature stop codon at the position 1183 that reduces their maltotriose uptake (Vidgren et al., 2009). Moreover, the *MAL* loci are present in several subtelomeric regions, resulting in CNVs that affect the transport capacity of the strains (Brown et al., 2010). The impact of sugar assimilation on fermentation kinetics has been also demonstrated for the *SUC2* gene, encoding for the invertase, that can be present in numerous subtelomeric regions especially in bakery strains (Carlson and Botstein, 1983; Ness and Aigle, 1995). Besides their integrity and their copy number, α-glucoside transporters may be also differentially regulated. A documented example was provided in bakery strains having an nsSNP in the *MALx3* gene, which encodes for the transcriptional activator of the maltose permease and maltase (Higgins et al., 1999). This *leu243phe* substitution abolishes the glucose catabolite repression conferring constitutive maltose consumption. In a similar way, galactose is a carbon source also present in many industrial media (cheese whey, molasses, and lignocelluloses) (Bro et al., 2005). Mutations in *GAL80*, the repressor of the galactose utilization pathway, have been generated by adaptive laboratory evolution and lead to a constitutive activation of galactose consumption (Segrè et al., 2006).

Hexoses (fructose and glucose) transport is also modulated by CNVs and punctual mutations. ALE studies demonstrated that hexose transporter genes alike *HXT6/7* are found in numerous copies (Kvitek and Sherlock, 2011). The uptake of fructose, which is less assimilated than glucose (Berthels et al., 2004), is also affected by allelic variations. For instance, allelic variations in the major hexose transporter Hxt3p or in the uncharacterized protein Rbh1p have been reported (Guillaume et al., 2007; Salinas et al., 2012).

##### Glycerol Production/Consumption and the Modulation of Sugar-to-Ethanol Yield

Since glycerol represents the main metabolic by-product of the alcoholic fermentation, its consumption by microbial conversions has been evaluated in order to valorize the dramatic surplus of crude glycerol produced in the biofuel industry (Clomburg and Gonzalez, 2013). Favorable alleles enhancing the *S. cerevisiae* growth rate on glycerol as unique carbon source was identified by QTL mapping. Two genes were identified: *GUT1*, which encodes for a glycerol kinase (Swinnen et al., 2013), and *TAO3*, a scaffolding protein involved in cellular morphogenesis (Wilkening et al., 2014).

Alternatively, the genetic bases of the glycerol production were explored. In bioethanol industry, a reduction of glycerol production would enhance the ethanol yield, which is, therefore, a valuable trait. In this context, the glycerol/ethanol ratio variability was assessed in 50 strains, and this genetic variability was used for achieving QTL mapping programs (Hubmann et al., 2013a; Hubmann et al., 2013b). The *SSK1* *^E330N … K356N^* allele (Hubmann et al., 2013a) was identified; this recessive allele found in the parental strain CBS6412 leads to a half-truncated protein. Its integration in the genome of the industrial background “Ethanol Red” has a substantial impact, decreasing by 23% its glycerol yield. Further investigations in the same background allowed the identification of three other alleles affecting the glycerol yield. Genes involved are related to the glycerol pathway (*SMP1*, *HOT1*, and *GPD1*) (Hubmann et al., 2013b). The *GPD1* *^L164P^* allele produces the largest effect and shows epistatic relations with other two loci.

In contrast, a growing demand of strains showing a lower ethanol production is observed in the alcoholic beverage industry and especially in winemaking. This demand moves in the direction of public health policy and could be in part solved by the development of new strains (Kutyna et al., 2010; Dequin et al., 2017). The most explored route consists in redirecting a part of the glycolytic flux toward the production of glycerol since this compound is organoleptically neutral. Allelic variations in *GAT1*, *YFL040W*, *GPD1*, and *ADH3* impacting glycerol yield (Salinas et al., 2012; Tapia et al., 2018) were identified by the same laboratory in a wine context. However, the highest production observed did not modify the sugar-to-ethanol conversion in a significant manner.

#### Acetic Acid Production

Acetic acid can be produced by spoilage microorganisms but is also produced by yeast during the alcoholic fermentation (Vilela-Moura et al., 2010). Acetic acid production level is a quantitative trait that varies across isolates (Giudici and Zambonelli, 1992; Marullo et al., 2004). In Lambic beers and some other sour beer styles, acetic acid can be a desirable component that contributes to the complexity of the flavor and aroma profile. However, in beverage industries, acetic acid generally has a negative impact on the organoleptic quality especially for wine (Ribéreau-Gayon et al., 2006). Therefore, the allelic variants controlling acid acetic production during fermentation were mostly identified in a winemaking context.

The major gene involved in acetic acid production in wine fermentation is *ALD6*, which encodes an aldehyde dehydrogenase (Remize et al., 2000; Saint-Prix et al., 2004). Depending on the yeast isolate, this gene is differentially expressed leading to various acetic acid production levels. Two SNPs in the promoter region of the *ALD6* gene were linked to this variation in an enological context (Salinas et al., 2012). Due to its role in the redox balance homeostasis, the acetic acid production level could also correlate with cell growth. A deleterious mutation in the catalytic core of the asparaginase *ASP1* gene was linked to the slow assimilation of asparagine in synthetic must. This affects the cell growth, causing overproduction of acetic acid in asparagine-rich media (Marullo et al., 2007a). More relevant are the premature stop codons found in the *YAP1* gene (YAP1^Q541X^ and YAP1^Q573X^). Those mutations were isolated by screening cerulenin-resistant mutants of the wine starter PDM. They dramatically decreased (−30%) acetic acid production during the fermentation of different grape juices (Cordente et al., 2013). Yap1p is a transcriptional factor involved in oxidative stress, and the metabolic link between these mutations and the phenotype was not perfectly understood. Interestingly, these mutants showed a high alcohol dehydrogenase activity with an increased production of acetate esters that could reduce the acetic acid production (see below).

#### Fermentation Efficiency

Several allelic variants affecting the overall fermentation rate in diverse industrial contexts have been identified. In the bakery context, a noteworthy trait is the *FIL* phenotype that consists in losing stress resistance when the alcoholic fermentation is initiated. Some strains show a recessive *fil* phenotype (fermentation-induced loss of stress resistance) and conserve a suitable resistance to heating and freezing even if they are collected in exponential growth phase during alcoholic fermentation (Thevelein et al., 1999). This particular feature can be obtained by using specific mutations in the gene *CYR1* (adenylate cyclase) at the position G1682L or in the gene *GPR1* (G-protein coupled receptor). The *fil* phenotype conferred is particularly relevant for obtaining full active strains for the fermentation from frozen doughs or active dry yeast.

Other allelic variants affecting the fermentation rate itself were identified in high-gravity matrices such as sake and wine. Genes impacted may encode for unexpected functions like the glycogen debranching protein (Gdp1p) (Cubillos, 2016). Interestingly, the positive allele of the *GPD1* gene is located in both the promoter and coding regions. Although the physiological mechanisms of these natural variants are not particularly linked to alcoholic fermentation, the allele of a wine-related strain (WE) confers a faster fermentation. Other mechanisms of adaptation were found by investigating the performance of wine yeast in the second fermentation that took place in locked bottles (*méthode champenoise*). Fermentation kinetics were measured by following the CO_2_ pressure rise inside the bottle. Two genes encoding for components of the plasma (*PMA1*) and vacuolar (*VMA13*) membrane ATPases were identified. Positive alleles provide a faster fermentation kinetics in low pH conditions (2.8), which is a particular feature of sparkling wines. The same authors identified two other genes related to osmotic regulation (*MSB2*) and multidrug resistance (*PDR1*) (Martí-Raga et al., 2017). Interestingly, transcriptional regulators of this multidrug resistance family (PDR network) have been also linked to the fermentation resistance in a sake-brewing context. By applying a drug resistance screening, different alleles of the transcriptional factors *PDR1* (M308I) and *PDR3* (L950S, G948D, and G957D) were isolated. These alleles drastically improved the fermentation efficiency of sake strain, allowing the production of more than 21% (v/v) of ethanol in industrial trials (Mizoguchi et al., 2002).

Another relevant industrial property of fermenting yeast is the length of the lag phase that could be particularly critical for achieving the inoculation of non-sterile musts. The genetic determinism of the lag phase has been partially elucidated in wine fermentations by performing a QTL mapping between two wine-related strains (Zimmer et al., 2014). In this work, a major QTL explaining relevant differences in the lag phase duration (more than 24 h) was identified. In this specific case, the molecular cause of phenotypic discrepancy is due to a reciprocal translocation event (XV-t-XVI) involving the gene *SSU1*, which encodes for a sulfite pump. This gross chromosomal rearrangement increased the expression level of *SSU1* in the parental strain GN that achieves a rapid fermentation start in synthetic grape juice containing SO_2_. This work illustrates an interesting case of phenotypic convergence since two other independent chromosomal rearrangements, VIII-t-XVI (Pérez-Ortín et al., 2002) and inv-XVI (García-Ríos et al., 2019), targeting *SSU1* and conferring SO_2_ resistance were identified. Recently, the pleiotropic effect of these translocations has been demonstrated by seeking for QTLs that interact with environmental conditions (Peltier et al., 2018). Depending on the nature of the grape juice and the amount of free SO_2_ in the medium, the translocations associated with *SSU1* may impact the production of SO_2_, lag phase, and fermentation rate.

### Resistance to Toxins and Stresses

Industrial applications are characterized by a broad set of stresses such as osmotic, temperature, ethanol, pH, nutrient limitation, and presence of various toxins that affect the yeast cell growth and viability (Bauer and Pretorius, 2000; Gibson et al., 2007; Zhao and Bai, 2009; Sicard and Legras, 2011). Each particular stress activates specific and general stress responses, ensuring a better physiological adaptation (see Gasch, 2003). Although most of these stresses are common, each biotechnological process recreates particular conditions explaining the emergence of yeast strains adapted to each specific process (Sicard and Legras, 2011; Albertin et al., 2011). In this section, we point out natural genetic polymorphisms in 75 genes that impact the yeast resistance to several types of stress commonly found in biotechnological applications (**Table 1** and **Table S1**).

#### Ethanol Tolerance

Ethanol accumulated during the fermentation impacts negatively the more sensitive strains, impairing the fermentation completion. Stuck fermentations affect the ethanol production yield and also the microbiological stability of beverages due to the presence of residual sugars. The selection of ethanol tolerant strains constitutes a real challenge in particular for sake and wine production where high concentrations are reached (respectively, 20% and 17%). Several hundred genes associated with ethanol tolerance were identified by functional genetics (see Ma and Liu, 2010; Snoek et al., 2016, for a review); however, identifications of causative SNPs are more rare.

Among the *S. cerevisiae* species, the sake strains demonstrate the highest capacity of ethanol accumulation with concentration reaching approximately 20% (v/v). Several genetic causes explaining this characteristic have been identified by comparing the transcriptome between sake and laboratory strains during sake fermentation (Wu et al., 2006; Shobayashi et al., 2007). Sake strains carry deleterious alleles in genes involved in the stress response and quiescent phase entry (*RIM15 *and *MSN2*/*MSN4*) (Watanabe et al., 2011, Watanabe et al., 2012). In addition, they lack the *PPT1* genes involved in the heat shock stress response (Noguchi et al., 2012). These mutations private to the sake group (**Table S3**) may explain their high ethanol accumulation capacity.

Global transcription machinery engineering (gTME) was used to generate strains more tolerant to ethanol. One of the targeted genes is *SPT15* encoding for a TATA-binding protein associated with ethanol tolerance (Alper et al., 2006). The random mutations generated globally modify the yeast transcriptional response, providing mutants with a higher ethanol tolerance (Yang et al., 2011). This strategy allowed the isolation of two *SPT5* haplotypes that enhanced the ethanol production from 8% to 10% for the yeast strain L3262. Those alleles also confer a better tolerance to hyperosmotic stress (Kim et al., 2013), another character highly desirable for high-gravity brewing (HBV) fermentation.

Besides the differential activation of these transcriptional pathways, numerous QTNs impacting ethanol tolerance have been successfully identified by reverse genetics. Since ethanol exerts a toxic effect, resistant strains can be readily screened by applying selective media where only a part of the population overcomes a desired threshold. Historically, adaptive laboratory evolution has been successfully used (Chen et al., 2010; Stanley et al., 2010; Avrahami-Moyal et al., 2012; Voordeckers et al., 2015). By increasing ethanol concentration in turbidostatic cultures, causative SNPs in the *SSD1* and *UTH1* genes were identified (Avrahami-Moyal et al., 2012). The selective pressure imposed (up to 8% ethanol) and the laboratory background used in this study are, however, a bit far from the conditions met in industrial fermentations. In similar conditions, a long-term evolution experiment was applied for 200 generations in six independent bioreactors (Voordeckers et al., 2015). Ploidy level changes and CNVs were mainly observed. Some QTNs were also identified: alleles *VPS70^C590A^*, *PRT1^A1384G^*, *IAI11^G479T^*, and *MEX67^G456A^* conferred an adaptive advantage by impacting remarkably diverse molecular functions such as mRNA export (*Mex67p*), vacuolar protein sorting (*Vps70p*), and protein synthesis (*Prt1p*).

QTL mapping approaches were also used for exploring the tolerance to ethanol with concentrations closer to industrial conditions (up to 20%) (Swinnen et al., 2012a; Pais et al., 2013; Duitama et al., 2014). In these studies, segregants were screened on YPD plates with different ethanol concentrations and genotyped using a bulk-segregant analysis (BSA). RHA validated the effect of five genes involved in the ethanol tolerance: *MKT1*, *SWS2*, *APJ1*, *ADE1*, and *KIN3*. The negative impact of the S288c allele of *MKT1* was found in both studies and is due to a rare deleterious allele (minor allele frequency (MAF) = 0.1%) that has a strong impact on many characters of the laboratory stain (Lee et al., 2009). This type of defective allele is not present in industrial strains and cannot be used for their improvement. However, pairs of positive alleles (*KIN3* and *ADE1*) (Pais et al., 2013) and (*APJ1* and *SWS2*) (Swinnen et al., 2012a) were brought by parental strains isolated from sake and biofuel, respectively. For *APJ1*, a clear ASE effect was observed demonstrating that the expression of *APJ1* seems to be deleterious for high ethanol tolerance (Swinnen et al., 2012a).

#### Cold and High Temperature Tolerance

Each industrial process is characterized by the application of temperatures that often meet yeast physiological limits. In biofuel industry, distillery yeast must tolerate a high temperature (up to 40°C) in order to ferment in association with enzymatic cocktails used for the saccharification (Olofsson et al., 2008). The wine or sake industry imposes milder conditions with temperature that does not exceed 35°C (Marullo et al., 2009). Furthermore, cold temperature tolerance (6–15°C) can be required especially in brewery and white wine context. Finally, the bakery process imposes an extremely broad range of temperature by applying freeze/thaw cycles.

The first quantitative genetics study in yeast focused on high-temperature growth (HTG) phenotype, which consists in measuring the colony size after a 48-h culture at 41°C (Steinmetz et al., 2002). The dissection of a major QTL by RHA allowed identification of three genes (*MKT1*, *END3*, and *RHO2*) explaining the quantitative phenotypic variation between the laboratory strain S288c and a clinical isolate YJM145. The identification of causative SNPs was achieved few years later (Sinha et al., 2006) by using site-directed mutagenesis. RHO2 causative SNP is located in the 3′UTR region, which constitutes a rare example of polymorphism outside the coding sequence. A supplemental gene impacting this phenotype,* NCS2*, was finally identified by using a backcross strategy in order to eliminate the effect of the main segregating QTL (Sinha et al., 2008). Using a bulk sequencing analysis strategy (Yang et al., 2013) confirmed the deleterious inheritance of S288c alleles for *NCS2 *and* MKT1*. It also identified two other causative genes (*PRP42* and *SMD2*) encoding for proteins belonging to the same spliceosome complex, suggesting complex epistatic relations. Indeed, the authors demonstrated that the thermotolerant alleles were *PRP42* ^S288c^ and *SMD2* ^MUCL2817^. However, when the *PRP42* ^S288c^ was introduced in the MUCL2817 genetic background, no additional effect was observed.

By implementing panmictic crosses (advanced intercross lines) between two natural isolates (West African and North American strains), Parts et al. (2011) underlined the role of RAS/cAMP signaling pathway for high-temperature growth by demonstrating the impact of the two paralogues (*IRA1* and *IRA2*) encoding for the RAS inhibitor proteins. Similar phenotypes were also elucidated at the gene level and concern the pyruvate kinase protein Cdc19p (Benjaphokee et al., 2012) or the E3 ubiquitin ligase *Rsp5p* (Shahsavarani et al., 2012).

All these studies investigated the temperature tolerance of *S. cerevisiae* strains by evaluating their growing capacities. However, in industrial fermentation, the deleterious effect of high temperature is generally coupled with many other stressful conditions including high ethanol content (Marullo et al., 2009; Mitsumasu et al., 2014), presence of toxins (Taherzadeh et al., 2000; Hasunuma et al., 2011), and sterol or nitrogen depletion (Bely et al., 1990; Marullo et al., 2009).

In a recent study, two genes, *OYE2* and *VHS1*, impacting the fermentation rate at high temperature were identified in a winemaking context (Marullo et al., 2019). In both cases, an SNP was generating a codon-stop insertion impairing the completion of the fermentation above 30°C. Interestingly, for the *VHS1* gene, the truncated protein of 371 amino acids confers a more efficient fermentation above 30°C. The recently documented function of *Vhs1p* (Simpson-Lavy et al., 2017) allows establishing a link between respire–fermentative switch and the fermentation efficiency at high temperature.

Alternatively, some industrial processes (especially food-related fermentations) impose cold and negative temperatures that may have drastic consequences on yeast fitness. If the physiological and molecular mechanism of cold tolerance has been understood (Gunde-Cimerman et al., 2014), few studies have investigated the natural genetic variation impacting cold adaptation.

In a recent work, genes related to lipid remodeling in the plasma membrane or mitochondrial metabolism were identified (García-Ríos et al., 2017). The phenotype investigated was the specific ability to growth at low temperature (15°C) in a synthetic grape juice. The selective genotyping of a pool of progenies derived from two wine-related strains (P24 and P5) was used. The impact of the *FPK1* gene encoding for a protein kinase that regulates phospholipid translocation and membrane asymmetry was clearly demonstrated. A substitution *R520K* in the P24 strain seems to be deleterious for growth at 15°C but not at 28°C. Interestingly, most of the QTLs mapped are located in subtelomeric regions of chromosomes XIII, XV, and XVI. Many of tested genes affect the time to achieve the fermentation, underlining the role of mitochondrial proteins (*QCR2* and *PET494*), oligopeptide transporter (*OPT2*), and aquaporin (*AQY1*), which seem particularly important for maintaining a fermentation activity at low temperature. The same team carried out an evolution experiment using the P5 strain and identified an nsSNP in the gene *GAA1*, which encodes for a protein belonging to the GPI–protein transamidase complex. The introduction of a threonine at the position 108 of this protein enhances the growth fitness at 12°C, suggesting the involvement of mannoproteins in cold adaptation (García-Ríos et al., 2014).

The impact of yeast aquaporins encoded by *AQY1* and its paralogue *AQY2* was also implicated in freezing and osmotic tolerance. Many loss-of-function mutations in these genes are present in the *Saccharomyces cerevisiae* wild population (Will et al., 2010). Moreover, differential expression was described for *AQY2* and are due to polymorphisms in the promoter region (Fay et al., 2004). In a bakery context, it is well known that functional and overexpressed aquaporins enhance freezing tolerance, while non-functional ones promote osmotic tolerance (Tanghe et al., 2002; Will et al., 2010). This suggests that *AQY* genes are submitted to balancing selection (Will et al., 2010).

Other mutations enhancing the proline accumulation also improve freezing resistance. The intracellular storage of this amino acid confers resistance to many stresses including freezing, desiccation, oxidation, and ethanol and enhances the fermentation kinetics (see Takagi, 2008 for review; Kitagaki and Takagi, 2014). Mutations in the *PRO1* were generated by either the selection of proline-analogue resistant mutants (Takagi et al., 1997) or by PCR random mutagenesis (Sekine et al., 2007). These mutations desensitize Pro1p against the feedback inhibition exerted by proline. Disruption of *PUT1* involved in the proline degradation pathway also enhances freezing resistance (Takagi, 2008) and was successfully combined with *Pro1p* (*D154N*) or (*I150T*) mutations in a self-cloned diploid strain (Kaino et al., 2008). Interestingly, a similar combination of a *Pro1p* mutation with an *Mpr1p* variant (*F65L*) enhances both freezing and air-dry resistance (Sasano et al., 2012).

#### Osmotic Stress

Most of the industrial processes involving *Saccharomyces* species are characterized by a high sugar content that goes hand in hand with a severe osmotic stress. Improving the resistance to osmotic pressure is therefore essential for achieving high-gravity ethanol fermentations. The osmotic stress response in yeast has been widely investigated (Tamás and Hohmann, 2003), and several mutations affecting osmotolerance have been intensively identified (Hohmann, 2002). Natural or induced mutations have been reported in many distinct pathways, including Hog1p activation (Pbs2p*^K389M^* (Reiser et al., 2000) and *Sln1p* *^P1148S/P1196L^* (Fassler et al., 1997)), proline accumulation (*PRO1*), and water efflux (*AQY1-2*) (see above). More recently, a QTL mapping study identified genetic causes of osmotic shock associated with very-high-gravity ethanol fermentations using the SGRP-X4 design (Greetham et al., 2014). The alleles *RCK2* ^Q113/S456^ of the wine strain DBVPG6765 enhance osmotolerance. This kinase performs a regulatory role in the *Hog1p* pathway and is involved in osmotic stress response since its overexpression improved growth in high osmotic conditions (Teige et al., 2001).

#### Resistance to Toxins

In the last decade, the biofuel industry has moved from the first- to second-generation production. This technological progress consists in transforming pentose sugars present in the plant cell walls in ethanol, in addition to hexoses. This additional step required lignocellulose pre-treatments that release aromatic and acidic compounds that are detrimental to the growth of *S. cerevisiae* (Palmqvist and Hahn-Hägerdal, 2000; Klinke et al., 2004). First of all, yeast has to face acetic acid and other weak acids that decrease the cytosolic pH, inhibit growth, and remodel gene expression (see Palma et al., 2018, for a review).

In a QTL mapping study, the *COX20* ^Q9R^ allele of the cytochrome *c* oxidase assembly factor has been identified (Greetham et al., 2014). This allele confers sensitivity to mild concentrations of acetic acid and other weak acids (formic and levulinic) likely linked with the programmed cell death response. In a similar way, the fermentation kinetics in a culture medium spiked with acetic acid was measured for a progeny population derived from a cross between a biofuel strain (ethanol red) and an acetic acid-resistant strain JT22689. A major QTL was mapped on chromosome XVI and was associated with an nsSNP impacting the coding sequence of the gene *HAA1*. The resistant strain carries a unique polymorphism at the nucleic position 1571 that generates a single amino acid change S505N (Haa1*) (Meijnen et al., 2016). This transcription factor plays a central role in the *S. cerevisiae* adaptation and tolerance to weak acids (Palma et al., 2018). The Haa1* allele activates the expression of plasma membrane acetate exporters Tpo2p and Tpo3p. Interestingly, another punctual mutation (*S135F*) promoting acetic acid resistance has been identified by a gTEM approach (Swinnen et al., 2017).

In a second round of QTL mapping performed in a panmictic population (inbreeded lines), four other causative genes have been detected (*CUP2*, *VMA7*, *GLO1*, and *DOT5*) with the positive contribution of the acetic acid-resistant strain JT22689 (Meijnen et al., 2016). Interestingly, *CUP2* is a paralogue of *HAA1*, suggesting that the expression of acetate transporter is a preferential target of acetic acid resistance. The *VMA7* gene is involved in the vacuolar-pH homeostasis and was previously linked to acetic acid resistance. The last two genes, *GLO1* and *DOT5*, were never linked to weak acid resistance. They are related to osmotic and oxidative stresses, respectively.

Adaptive laboratory evolution experiments were also implemented for obtaining mutations enhancing the acetic acid resistance. After ∼50 transfers of alternative microaerobic batch cultivations (with and without acetic acid), five independent evolved cultures showing a strong resistance to acetic acid were obtained. Four causal mutations in the genes *ASG1*, *ADH3*, *SKS1*, and *GIS4* were identified by genome sequencing and validated by allele replacement (González-Ramos et al., 2016). Three of them (*ADH3*, *SKS1*, and *GIS4*) were not previously associated with acetic acid tolerance, providing new clues for understanding this complex trait. Interestingly, other genes related to weak acid resistance were found in a winemaking context. Although acetic acid does not impact cell growth in winemaking, a relevant allele impacting the weak acid resistance has been identified by eQTL mapping (Brion et al., 2013). By analyzing the whole-genome expression profile of 44 progeny clones (BY × 59A cross), these authors identified five nsSNPs in the coding sequence of *WAR1* gene. This gene encodes for a transcription factor that controls the expression of a plasma membrane ABC transporter responsible (Pdr12p) for organic acid efflux. Allele swapping experiment demonstrated that the *WAR1* ^59A^ wine yeast allele increases the expression level of *PDR12* and enhances the sorbic acid resistance.

Cellulose and hemicellulose hydrolysis released furfural and 5-hydroxy-methyl-furfural (HMF) that have a strong toxic effect on yeast growth and fermentation (Palmqvist and Hahn-Hägerdal, 2000). To date, only one allele of the main alcohol dehydrogenase (*ADH1*) favoring resistance to HMF was identified in an industrial isolate. The protein sequence reveals multiple amino acid polymorphisms close to the substrate binding pocket (S109P, L116S, and Y294C). This allele has a NADH-dependent HMF reductase activity, which is not present in any other strains and allows reduction of HMF in a nontoxic form (Boaz et al., 2008).

Toxin resistance of lignocellulosic raw material has been recently investigated by two other original approaches. First, a sophisticated strategy combining mutagenesis, genome shuffling, and phenotypic selection was implemented in order to isolate mutations, enhancing resistance to hardwood spent sulfite liquor (Pinel et al., 2015). Among a dozen of putative SNPs identified by whole-genome sequencing, these authors demonstrated that a single mutation in the coding sequence of the gene *UBP7* (2466 T > A) conferred a better tolerance to this medium. Second, a GWAS linked 76 SNPs with growth traits measured in complete hydrolysates spiked with a toxin cocktail (Sardi et al., 2018). The association was performed by keeping SNPs from having a minor allele frequency greater than 2% in a collection of 165 fully sequenced *S. cerevisiae* strains. The effect of allelic variants in the *LEU3*, *MNE1*, and *SAP190* genes was validated by RHA in different genetic backgrounds (Sardi et al., 2018).

### Organoleptic Properties

**Figure 6:**
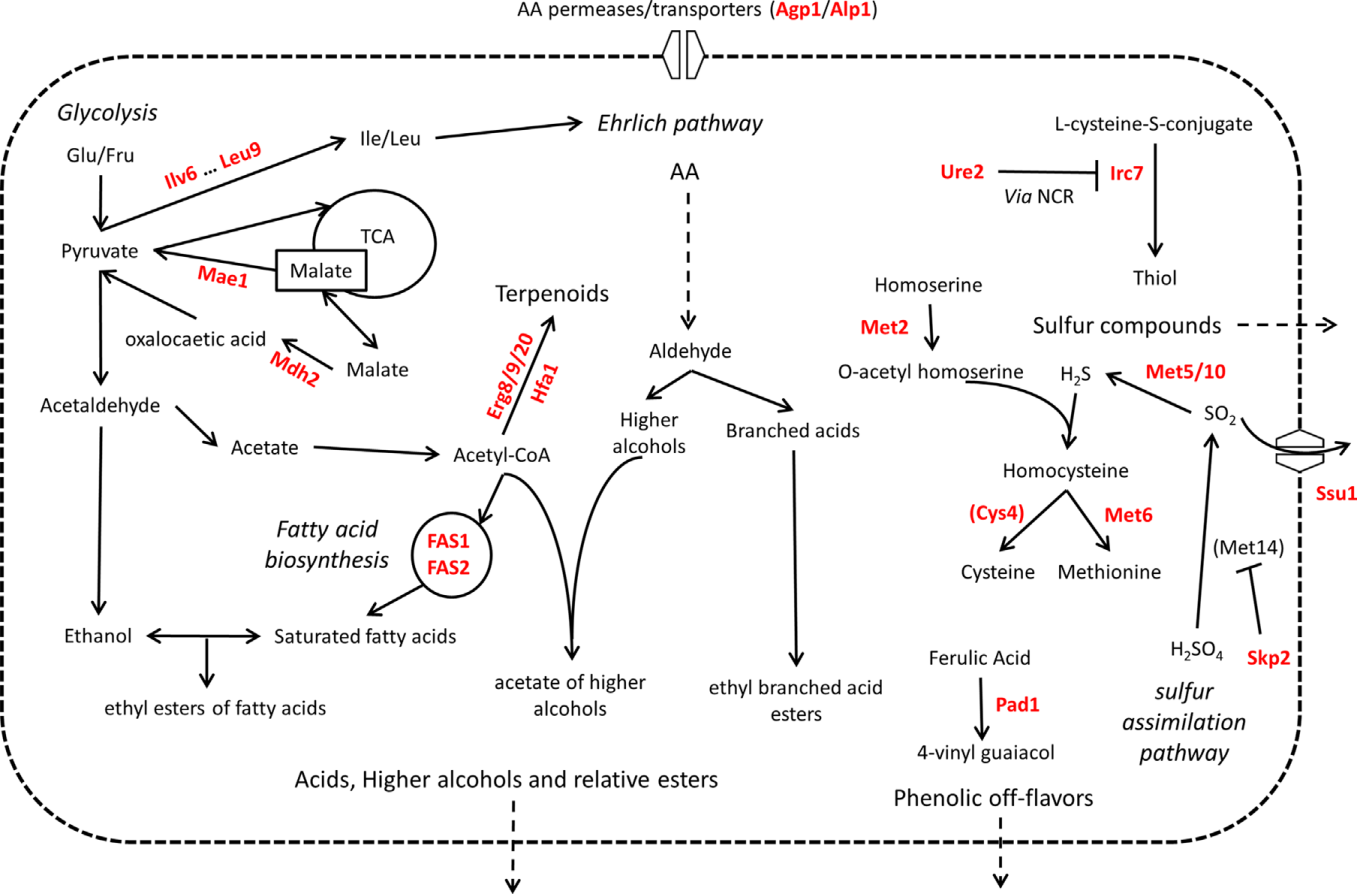
Overview of QTG involved in organoleptic compound production. Proteins highlighted in red are coded by allelic variants that were experimentally validated for their contribution to variation in organoleptic compound production in *S. cerevisiae*. Indirect relationships are shown in dashed lines. Proteins are between brackets when only the protein of interest of a pathway is shown. TCA = tricarboxylic acid cycle. NCR = nitrogen catabolite repression of transcription.

When they are used in food-related fermentation processes, the fermentation efficiency is not the only technological property desired. Yeast strains are also selected depending on their impact on the composition of several organoleptic compounds. In this last subsection, we reviewed the role of 48 genes and their relative QTNs that influence the organoleptic quality of beverages (**Figure 6**).

#### Higher Alcohols and Esters *de Novo* Synthesis

Higher alcohols and esters constitute groups of volatile compounds that are *de novo* produced during the alcoholic fermentation. Their organoleptic impact in fermentation beverage has been widely reviewed (Mason et al., 2000; Sumby et al., 2010), and the main genes and enzymatic activities controlling their biosynthesis have been identified (Malcorps et al., 1991; Verstrepen et al., 2003a; Saerens et al., 2006). Some nsSNPs in the sequence of these enzymes modulate the biosynthesis of these compounds. For example, the brewing yeast strains expressing a long form of the *ATF1* genes (*LgAFT1*) produce more acetate esters of higher alcohols (Verstrepen et al., 2003a). *ATF1* is also responsible for the majority of acetate ester production, an undesirable compound with a solvent-like off-flavor (Verstrepen et al., 2003b). To identify genetic factors responsible for the remaining acetate ester production, a QTL mapping study was carried out with or without *ATF1* deletion in parental strains (Holt et al., 2018). QTNs in *EAT1* and *SNF8* were identified, with rare alleles that prevent acetate ester production.

Moreover, many genetic variations affecting proteins in connection with esterification reactions were described. They concern the Ehrlich pathway, the acetyl-CoA production, and the lipid biosynthesis. For example, allelic variation in *ILV6* gene stimulates the production level of 2-methyl-propyl acetate by enhancing the biosynthesis of α-ketoisovalerate, its related precursor (Eder et al., 2018). The activity of the FAS complex (fatty acid synthase) has a direct effect on the biosynthesis of many esters. This complex of two proteins (Fas1p and Fas2p) synthesizes fatty acid by the repeated condensation of malonyl-CoA and acetyl-CoA. Since medium fatty acids (C6–C12) are the precursors of ethyl esters, their intracellular concentrations are directly linked to those of ethyl esters. Moreover, the activity FAS complex regulates the cytoplasmic acetyl-CoA pool; this metabolite is the substrate alcohol acetyl-CoA transferase (Atf1p and Atf2p) that produces acetic esters of higher alcohols. Therefore, mutations in the FAS complex affect both acetic and ethyl ester biosynthesis. For instance, ethyl esters biosynthesis in sake (i.e., ethyl-caproate) is modulated by the *fas2* ^G1250C^ defective allele that could be easily obtained due to their resistance to cerulenin (Aritomi et al., 2004). Interestingly, natural allelic variants in the *FAS1* gene explain a similar phenotypic discrepancy in a winemaking context (Eder et al., 2018). Other allelic versions of the *FAS2* gene alter the acetylation of higher alcohols like phenyl-ethanol acetate and isoamyl-acetate (Trindade de Carvalho et al., 2017).

The production level of esters and higher alcohols is also impacted by amino acid uptake. Indeed, several nsSNPs in the amino acid transporters encoded by the *ALP1*, *AGP1*, and *AGP2* genes control the phenotypic variance of acetate esters of higher alcohols observed among a large progeny (Eder et al., 2018). The same authors identified more distant enzymatic activities impacting the intracellular pool of acetyl-CoA (*SIR2*) or pyruvate (*MAE1*) that are building blocks of ester precursors. The framework between these precursor pathways and the esterification reactions shed light on the complex determinism of these aromas. The perturbation of folic acid biosynthesis has been also reported to modulate the production of phenyl-ethanol by identifying the deleterious allele *ABZ1 via* linkage mapping (Steyer et al., 2012). Finally, the deleterious mutation TOR1^E216*^ affecting the regulatory protein Tor1p has been also reported as reducing ester production by an unknown mechanism (Trindade de Carvalho et al., 2017).

#### Sulfur Compounds

Volatile sulfur compounds are important contributors to the flavor of many foods. These molecules are characterized by a low sensory detection threshold due to the high volatility of sulfur atoms. During alcoholic fermentation, *Saccharomyces cerevisiae* releases different classes of volatile sulfur compounds that could be *de novo* produced or bio-converted from precursor molecules. Although present at very low concentration levels (close to ppb), differences within strains strongly contribute to the quality of the final product. A key node of sulfur compound production is centered on the sulfite reductase enzymatic activity involved in the reduction of sulfate into sulfide ions, which ensure the incorporation of a sulfur atom in both methionine and cysteine. A leak of sulfide ions during alcoholic fermentation results in the overproduction of hydrogen sulfide (H_2_S) and high undesirable off-flavors in beverage industry (Swiegers and Pretorius, 2007).

Several approaches were used to identify genetic variants that might decrease H_2_S production. By screening an EMS-mutagenized population, several alleles reducing H_2_S production were found in the *CYS4*, *MET6*, *MET5*, and *MET10* genes (Linderholm et al., 2006; Cordente et al., 2009; Linderholm et al., 2010). A linkage analysis revealed natural genetic variations in this pathway affecting the *MET1* ^(^ *^A458T^* ^,^ *^T511I^* ^,^ *^G687D^* ^,^ *^E805K^* ^)^, *MET2* *^R301G^*, and *MET5* *^V288X^* genes that strongly modulate the H_2_S production during alcoholic fermentation (Huang et al., 2014). Interestingly, the *MET2* *^R301G^* allele was independently detected by another QTL mapping study as an enhancer of SO_2_ production (Noble et al., 2015) and is quite frequent in wine European group (**Table S2**). This finding highlights the fact that a reduced H_2_S production is often coupled with a high SO_2_ production as reported for *MET2* (Noble et al., 2015), *MET5* (Cordente et al., 2009), and *MET10* (Cordente et al., 2009; Huang et al., 2014). More interestingly, Noble et al. identified beneficial alleles in the *SKP2* gene that reduce both the SO_2_ and H_2_S productions. This protein encodes an F-box factor that regulates the abundance of Met14p involved in the first steps of the sulfate reduction pathway as previously demonstrated in a brewery context (Yoshida et al., 2010). By introducing an appropriate allelic variant (*T357I*) using a backcross strategy, the authors demonstrated that wine strains producing low SO_2_ amount can be readily selected (Blondin et al., 2014).

Another route for reducing the SO_2_ production consists in selecting strains devoid of a translocated *SSU1* allele (either XV-t-XVI or VIII-t-XVI; see Fermentation Efficiency. Since the *Ssu1p* transporter pumps the SO_2_ outside the cell, the less active allelic forms (non-translocated) reduce the concentration of this toxic compound in the fermented matrices. Although non-translocated alleles are not suitable in white grape juice fermentations due to the high level of free SO_2_, this feature can be used for red-grape juice fermentation (Peltier et al., 2018).

Sulfur volatile compounds may also contribute positively to the aroma of fermented beverages. One of the most achieved examples is given by the bioconversion of volatile thiols (4MMP, 3MH, and 3MHA) that contribute to the typicity of Sauvignon blanc wines (Tominaga and Dubourdieu, 2000). These powerful odorant molecules are derived from cysteinylated and glutathionylated precursors present in grape juice that are converted in volatile thiols by yeast β-lyases (Marullo and Dubourdieu, 2010; Coetzee and du Toit, 2012). The molecular dissection of this bioconversion led to the identification of different β-lyases. A relevant allele of *IRC7* explains most of variations in terms of 4MMP production (Roncoroni et al., 2011). The positive allele of *IRC7* was carried by a clinical *S. cerevisiae* isolate having a full-length protein of 400 amino acids able to convert the cysteinylated precursor in its relative aroma, whereas the 60-bp truncated form is not functional. The expression level of this gene is controlled by the nitrogen catabolite repression. Indeed, the use of *ure2* mutations is useful for enhancing the bioconversion rate of volatile thiols by enhancing the enzymatic activity of β-lyases (Thibon et al., 2008). Since non-GMO UV-mutations of *ure2* can be readily obtained (Marullo et al., 2008), appropriate *ure2* and *IRC7* *^400^* alleles were implemented for enhancing the volatile thiols bioconversion in order to select yeast strains expressing more intense notes of exotic fruits (Dufour et al., 2013).

#### Enhancement of Terpenoid Biosynthesis

Terpenoids constitute a wide class of natural molecules that can be produced by metabolic engineering for the production of antibiotics, anticancer, and other medicinal products and also for their aromas and fragrances (Ajikumar et al., 2008). These molecules can be synthetized by rerouting the sterol pathway that was first characterized in *Saccharomyces cerevisiae*. Natural genetic variations in the ergosterol pathway were identified for increasing the production of geraniol and linalool using a leaky FDP synthase (Erg20p) mutant (Chambon et al., 1991). This allele was introgressed into a wine yeast genetic background by repeated backcrosses (Javelot et al., 1991). The resulting strain has a satisfactory terpene production and aroma profile. Nevertheless, the alteration of the sterol synthesis pathway affected the ethanol tolerance, limiting its use in winemaking. A unique QTL mapping on terpene biosynthesis was carried out and allows the identification of one *PDR8* allele that increases nerolidol release in a synthetic media spiked with geraniol (Steyer et al., 2013).

#### Off-Flavor Reduction

Phenolic off-flavors (POFs), 4-vinyl phenol and 4-vinyl guaiacol, are unwanted compounds produced by yeast during beer fermentation and also in white wine production from phenolic acids present in the must. Therefore, a desirable characteristic of fermenting yeast is the phenotype POF- that is determined by the combined action of two genes localized in the subtelomeric region of chromosome IV. The *PAD1* gene encodes for a flavin prenyl-transferase, which catalyzes the formation of a prenylated cofactor required for the ferulic acid decarboxylase encoded by *FDC1* (Clausen et al., 1994; Mukai et al., 2010). A recent whole-genome sequencing project (Gallone et al., 2016) reveals that most of brewery strains and also some wine yeast strains have loss-of-function mutations in both *PAD1*(Q86*, Y98*, and W102*) and *FDC1*(K54*, Q154*, *c.495_496insA*, *c.864delA*, R309*, and W497*). These loss-of-function mutations are strongly correlated with the *POF-* phenotype (Mertens et al., 2017). Another *PAD1* allele (*PAD1* *^D213G^*) has been identified by QTL mapping (Marullo et al., 2007b) and was used for the selection of various white wine strains to avoid the production of these undesirable compounds (Marullo, 2009).

#### Flocculation Properties

Flocculation is the yeast cells’ capacity to co-aggregate and form flocs that can provoke cell sedimentation or the creation of a floating biofilm at the broth surface (Vidgren and Londesborough, 2011). These characteristics are important in the brewing industry where flocculation is necessary for yeast sedimentation at the end of the fermentation (Vidgren and Londesborough, 2011) or in winemaking where flor yeast development is essential to sherry wine production (Ossa et al., 1987; Fidalgo et al., 2006). Natural genetic variants that affect the flocculation capacity have been identified especially in the well-studied *FLO* genes that mostly encode for surface proteins.

The ability to form a floating biofilm is driven by an allelic version of *FLO11* in which a rearrangement within the central repeat domain leads to a more hydrophobic protein, and a 111-bp deletion in the promoter increases its expression (Fidalgo et al., 2006). *FLO11* not only concerns floating biofilm as the number of repetitions in the Flo11p central domain is also positively correlated to the flocculation strength (Wilkening et al., 2014). The same pattern is also found for Flo1p (Verstrepen et al., 2005). In the S288C genetic background, a premature stop codon in *FLO8* impairs flocculation. This gene encodes a transcription factor promoting the *FLO1* expression (Li et al., 2013). Conversely, a premature stop codon in the gene *SFL1* encoding a repressor of flocculation-related genes increases flocculation (Wilkening et al., 2014). Besides flocculation, the clumpiness of strains may be due to several allelic variants in the *AMN1*, *GPA1*, *RGA1*, *CDC28*, *FLO8*, *END3*, *IRA2*, *MSS11*, and *TRR1* genes, which have been identified in laboratory conditions. These non-sexual adhesion properties could play a critical role in industry (Pretorius, 2000).

### Moving from QTN Detection to Industrial Applications

In the previous section, we listed a catalogue of 147 genes with natural or induced genetic variants that impact quantitative traits of industrial relevance at least in one specific genetic background. Most of them correspond to alleles that could be considered as a promising reservoir of functional levers to modulate metabolic pathways and biological activity of the prime industrial microorganism, *Saccharomyces cerevisiae*. Since their number is steadily increasing, QTNs should have a profound impact for improving the selection methods of industrial strains in the future. These polymorphisms can be introduced in any desired yeast “*chassis*” by using GM (genetically modified) organisms as it was perfectly illustrated in the context of bioethanol production (Maurer et al., 2017). Depending on the industrial field and the local legal regulation, these QTNs may also be exploited by implementing more classical breeding strategies (Marullo et al., 2007b; Blondin et al., 2013; Dufour et al., 2013). Although the identification of causative SNPs is now a routine task, their efficiency for improving technological properties by marker-assisted selection (no GM) or allele replacement (GM) is still unpredictable. The two most critical issues that yeast researchers are facing are the incomplete penetrance/expressivity level of identified SNPs and the gene–environment interaction modulating their effect. These issues are well recognized in agronomical science and explain why numerous markers identified in academic studies failed to be translated in the domain of application (Xu and Crouch, 2008). In this last section, we documented examples in yeast and evaluate possible solutions to overcome them.

#### The Low Penetrance/Expressivity Issue

The penetrance is defined as the proportion of individuals in a population that express a phenotype associated with a specific genetic variation. Indeed, a genetic variation may affect the phenotype of some individuals but remains silent in other backgrounds. A practical illustration of this phenomenon is given by *MET10*, *EAT1*, and* SNF8* alleles, which do not have the same effect according to the genetic backgrounds (Linderholm et al., 2010; Holt et al., 2018). Therefore, deleterious modifications of a key enzyme in a well-defined pathway do not ensure a predictable phenotype. Low penetrance examples have been particularly well documented in the four parents cross designed by Ed Louis’s group, suggesting that most QTLs are largely cross dependent (Cubillos et al., 2013).

The simplest explanation is given by the fact that most of the strains carry loss-of-function alleles that are preferably detected by QTL analysis. Among the 284 nsSNPs reported, 8% confer a nonsense mutation. Moreover, 33% of the referenced missense mutations have a possible deleterious effect (see Genes and Polymorphisms Impacting Quantitative Traits of Industrial Interest. Therefore, up to 60% of QTNs reviewed have a MAF lower than 5%. This is confirmed by two recent studies showing that rare variants explain a disproportionately large part of the variation of quantitative trait in yeast (Bloom et al., 2019; Fournier et al., 2019). This high proportion is explained by the yeast life history with clearly defined subpopulations that have been evolved mostly by genetic drift in separated habitats (Liti et al., 2009; Peter et al., 2018). Although easy to identify, these loss-of-function alleles are scarcely relevant since they negatively impact the phenotype of interest. This is, for instance, the case of the *OYE2^Ser77sf^*, *ASP1^D142H^*, and *ABZ1^S288c^* alleles that drastically reduce fermentation performances in an enological context (Marullo et al., 2007a; Ambroset et al., 2011; Marullo et al., 2019). As a consequence, the opposite alleles that were defined as favorable have a very low impact since they are present in most of the other genetic backgrounds. To overcome this problem, different approaches were used for identifying minor QTLs (Brem et al., 2005; Sinha et al., 2008; Yang et al., 2013; Holt et al., 2018; Marullo et al., 2019). The segregation of the most impacting loci is eliminated from the segregants by its deletion in the hybrid, by selecting only a part of the segregants for linkage analysis or by generating new segregants with a targeted backcross. These strategies can help to identify QTLs with lower effects that better reflect the true differences between industrial strains.

In some cases, recessive, deleterious mutations have an industrial relevance when loss-of-function is associated with the desired trait as for the POF character (Marullo et al., 2007b; Mertens et al., 2017), and the *fil* phenotype (Thevelein et al., 1999). In natural isolates, most of these deleterious SNPs are masked in diploid progenitors since they are recessive. Therefore, before starting any QTL detection programs, it is relevant to discard meiotic progenies showing extremely “bad” phenotypes because they would transmit such deleterious SNPs.

Besides that, incomplete penetrance mostly results from genetic interactions between the causative allele identified and many other loci that impair its complete expressivity. Indeed, the Mendelian inheritance of a trait may turn out to be a quantitative in other genetic backgrounds (Hou et al., 2016). This is caused by the presence of modifier (epistatic) loci that modulate the expressivity of a major locus. Such epistatic loci have been identified in different species and in particular in yeast (Yadav and Sinha, 2018). First of all, epistasis concerns genes belonging to the same pathways and in particular between upstream regulator(s) and downstream effector(s). For instance, the favorable *GPD1^L164P^* allele had a reducing effect on glycerol yield for biofuel industry. This positive effect is only observed when two of its transcriptional factors (*HOT1* and *SMP1*) have the laboratory strain genotype (Hubmann et al., 2013b). Another example is given by the positive allelic combination of the fully active β-lyase *Irc7p^LT^* and the loss-of-function allele of the regulator Ure2p*^G181E^* that enhances the bioconversion of volatile thiols from cysteine conjugates precursors of grape juice (Dufour et al., 2013). In the same way, a strong positive epistatic interaction was found between *FLX1* and *MDH2* genes. Both genes play a role in the Krebs cycle, and the combination of *FLX1^SA^* and *MDH2^WE^* results in high levels of succinic acid during wine fermentation (Salinas et al., 2012). Besides these “obvious” metabolic connections, other interactions have been identified between functionally unrelated couples of genes. This is the case of *NCS2*–*MKT1* (Sinha et al., 2008) and *END3*–*RHO2* (Sinha et al., 2006), which strongly impacted HTG phenotype in the BY–RM11 cross. Once identified and understood, these epistatic relationships would be useful for dramatically enhancing a phenotype of interest by introducing suitable allelic combinations. Interestingly, a larger genetic modification such as aneuploidies (Sirr et al., 2015) may also play a modifier role and would be more difficult to control.

#### Genetic per Environment (GxE) Interactions

Environmental conditions represent the second major factor that drastically modifies the expressivity of genetic loci. It is noteworthy that among individuals of the same species, phenotypic plasticity is frequently observed (Veerkamp et al., 1994; Pigliucci and Kolodynska, 2002). These different non-parallel norms of reaction are due to GxE interactions. In yeast, the systematic research of genetic loci interacting with the environment has been achieved in several fundamental studies focusing on whole-genome expression level (Smith and Kruglyak, 2008; Gagneur et al., 2009), or growth traits (Gagneur et al., 2009; Bhatia et al., 2014; Wei and Zhang, 2017; Yadav et al., 2016) measured in divergent laboratory conditions. GxE interactions observed can be divided into two broad classes (Yadav and Sinha, 2018).

First, the effect of a locus may be environment-specific, reflecting the presence of a gene/allele adapted to a particular composition of the medium. This is often observed for traits related to a specific sugar transport (*MAL13*) (Bhatia et al., 2014) or for the adaptation to a specific toxin (*SSU1*) (Pérez-Ortín et al., 2002; Zimmer et al., 2014). Second, GxE interactions may explain phenotypic trade-offs illustrated by individuals showing a contrasted fitness across a pair of environments (Yadav et al., 2015; Wei and Zhang, 2017). These antagonistic effects are mostly due to the presence of one allele that has been positively selected in one environment but shows a negative effect in another one. Such contrasted responses are frequently observed when phenotyping is done in drastically different conditions such as respiratory versus fermentative conditions. In such cases, allelic variations of key regulatory genes have been detected in *IRA2* (Smith and Kruglyak, 2008) or *HAP1* (Wei and Zhang, 2017), which are both involved in metabolic pathway switches. Since most of these studies had a fundamental focus, the reaction norms were investigated in very divergent laboratory media in order to enhance the phenotypic plasticity. Therefore, it is likely that the importance of antagonistic effects claimed is certainly biased by the drastic physiological conditions imposed.

Pleiotropic QTLs showing large effects with both beneficial and negative impacts on species adaptation constitute an interesting case of GxE interactions. The description of pleiotropic QTLs/genes/SNPs has been achieved in several studies for *IRA2* (Yadav et al., 2015), *MKT1* (Deutschbauer and Davis, 2005; Fay, 2013), *CYS4* (Kim et al., 2009), *AQY1* (Will et al., 2010), and *SSU1* (Peltier et al., 2018). In some cases, these loci showed antagonistic effects and could reflect balancing selection since the alleles involved are found in similar frequency in natural populations. The aquaporin genes (*AQY1* and *AQY2*) are a notorious case of alleles under balancing selection with a possible effect in a bakery context. Indeed, loss of aquaporin reduces freeze–thaw tolerance but increases fitness in high-sugar environments, two conditions encountered in the bread industry (Will et al., 2010). Another case is given by the translocation XV-t-XVI that influences the expression level of the sulfite pump *SSU1* (Zimmer et al., 2014). Although very beneficial for initiating the alcoholic fermentation in the presence of SO_2_, yeast strains presenting this translocation have a reduced fermentation rate in the late steps of wine fermentation (Peltier et al., 2018).

In agronomy, QTL programs take into account the environmental effect by achieving phenotyping in various conditions. The first benefit of this strategy is the gain of power detection of minor QTLs. The second one allows the detection of robust loci having an effect whatever the environment is, which is an obvious asset for achieving marker-assisted selection. In a recent work, QTL mapping was achieved in three natural grape juices using two independent F1-cross derived from wine starters. The conditions applied represent extreme environmental conditions from an enological point of view (different grape juice colors, various amounts of sugar and nitrogen, and different micro-oxygenation conditions). In such conditions, up to 72% of the mapped QTLs have the same effect regardless of the environment. This observation suggests that when QTL mapping is carried out with genetic rootstock adapted to the specific industrial context, the GxE interactions are quite moderate. Therefore, most of the mapped QTLs would be robust in various enological environments (Peltier et al., 2018).

#### Selecting an Appropriate Genetic Background

**Figure 7:**
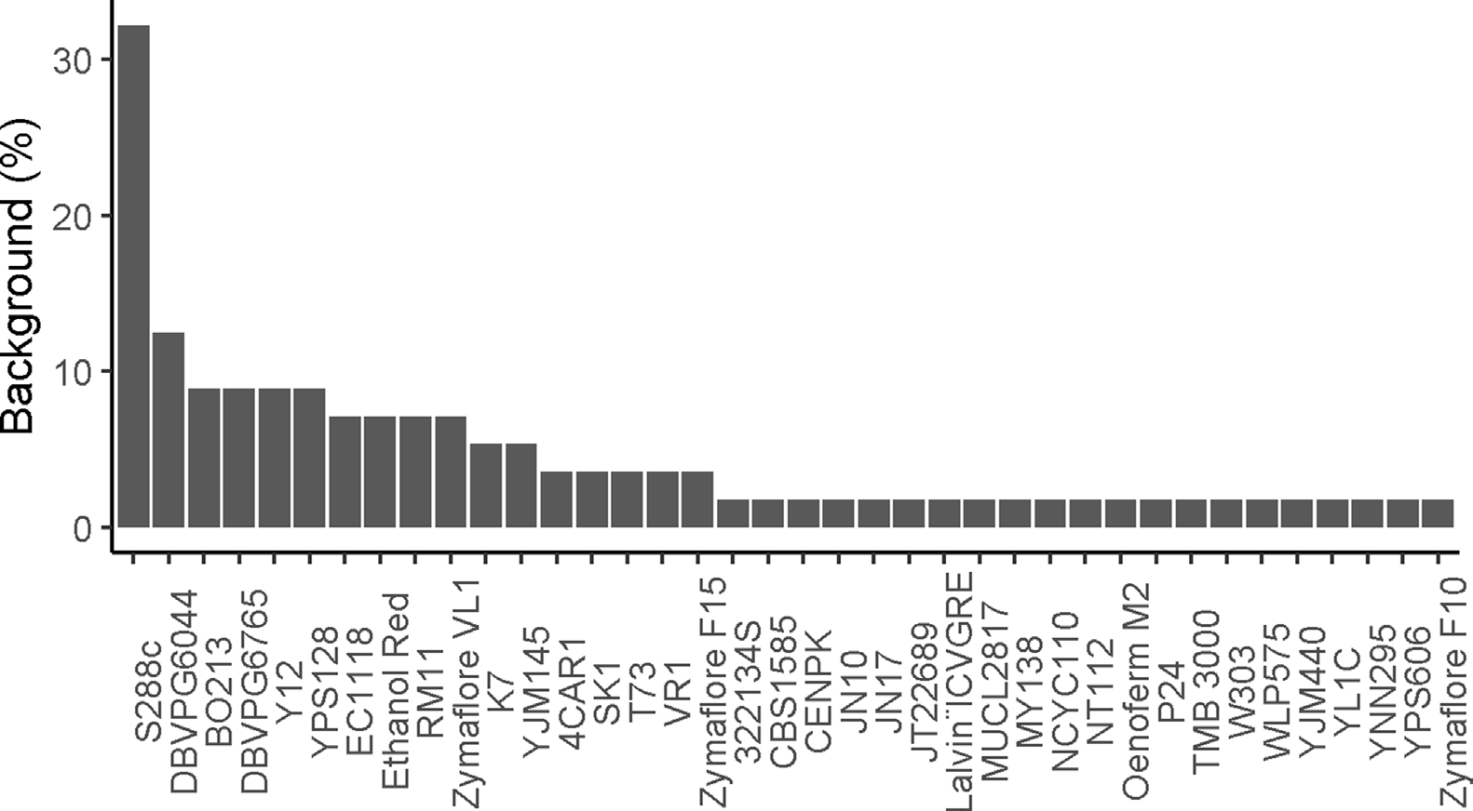
Proportion of genetic background used for QTL mapping studies. The percentage of time a genetic background was used in reported studies.

Until now, most QTNs were identified by using less than 20 yeast backgrounds (**Figure 7**). Usually, an elite strain with relevant features has been crossed with the laboratory background (S288c) or with one (or few) strains derived from another subpopulation. However, for achieving relevant research of biotechnological interest, the use of an “outgroup” partner may represent an important risk. This is particularly true for lab strains that have been cultivated for numerous generations in a controlled environment (growth on rich media at steady temperature). The use of various parental strains originated from distinct subpopulations (SGRP4X design) may have the same effect. Indeed, the NA, SA, WE, and WA strains belong to distinct clean lineages that have been evolved mostly by genetic drift in separated habitat. Although locally neutral in one environment, these alleles would have a deleterious effect on each other (Zörgö et al., 2013). Therefore, the selection of distant parental strains may enhance the chance to find QTNs with low expressivity by introducing a pool of not adapted alleles that *in fine* cause a drastic loss of fitness.

The cross of unrelated lineages also regroups a large pool of alleles that had never co-evolved together. This likely enhances epistatic interactions that have a negative impact on QTL expressivity. Then, we believed that the introduction of deleterious alleles must be prevented as far as possible. A rational way consists in using a set of strains adapted to the environment of interest. Indeed, by using such adapted strains, the pool of alleles submitted to positive selection would increase, while those having deleterious impact would be reduced. In most of the studies, parental strains showed very opposite trait values, which is not an obligation. In theory, two conditions are required for achieving linkage analyses. The first is to capture enough genetic polymorphisms for building a fine grain genetic map. This even can be obtained within strains of the same subpopulations. The second is a wide phenotypic segregation that is not necessary depending on parental traits. Recently, we demonstrated that the phenotypic and genetic distance within pairs of parental strains does not affect neither the phenotypic segregation nor the efficiency of QTL detection (Peltier et al., 2018). By extrapolation, the cross of two optimal strains with a sufficient phenotypic diversity would have the benefit to fix many positive alleles and allow the segregation of other QTLs that would also contribute positively to the phenotype.

## Concluding Remarks

This review provides a first compendium of QTNs of biotechnological interest belonging to the *Saccharomyces cerevisiae* species. This emphasizes the success of quantitative yeast genetics for identifying relevant natural (or induced) genetic variations that confers technological properties. The SNPs reported here will constitute a rich reservoir of genetic variations useful for improving the technological properties of industrial strains by using breeding or genome editing strategies. However, for bridging the gap between the identification of causative SNP and their routine exploitation, the complex architecture of quantitative traits needs to be better understood.

## Data Availability

The datasets analyzed for this study can be found in Peter et al. (2018).

## Author Contributions

EP and PM wrote the first draft of the manuscript. AF and JS wrote sections of the manuscript and reviewed and edited the original draft.

## Conflict of Interest Statement

EP and PM are employed by LAFFORT. The remaining authors declare that the research was conducted in the absence of any commercial or financial relationships that could be construed as a potential conflict of interest.

## Abbreviations

3′UTR, 3′ untranslated transcribed region; ALE, adaptive laboratory evolution; CNVs, copy number variants; eQTL, expression QTL; gTME, global transcription machinery engineering; GM, genetically modified; GWAS, genome-wide association studies; GxE, genetic per environment; HMF, 5-hydroxy-methyl-furfural; InDels, insertions/deletions; MAF, minor allele frequency; MAS, marker-assisted selection; nsSNP, non-synonymous SNP; sSNP, synonymous SNP; QTL, quantitative trait loci; QTG, quantitative trait genes; QTN, quantitative trait nucleotides; RHA, reciprocal hemizygosity assay; SNP, single-nucleotide polymorphisms.

## Supplementary Materials

Table S1 Description of the QTGs reported.

Table S2 Allelic frequencies of the QTNs reported.

Table S3 Distribution of the allelic frequencies among isolate origins.
